# Discovery of Nonretinoid Inhibitors of CRBP1: Structural and Dynamic Insights for Ligand-Binding Mechanisms

**DOI:** 10.1021/acschembio.3c00402

**Published:** 2023-09-15

**Authors:** Jacqueline Plau, Christopher E. Morgan, Yuriy Fedorov, Surajit Banerjee, Drew J. Adams, William S. Blaner, Edward W. Yu, Marcin Golczak

**Affiliations:** ^†^Department of Pharmacology, ^‡^Small Molecule Drug Development Core Facility, ^§^Department of Genetics, and ^∥^Cleveland Center for Membrane and Structural Biology, School of Medicine, Case Western Reserve University, 10900 Euclid Avenue, Cleveland, Ohio 44106, United States; ⊥ Department of Chemistry, Thiel College, Greenville, Pennsylvania 16125, United States; # Department of Chemistry and Chemical Biology, Cornell University, Ithaca, New York 14850, United States; ∇ Northeastern Collaborative Access Team, Argonne National Laboratory, Argonne, Illinois 60439, United States; ○ Department of Medicine, College of Physicians and Surgeons, Columbia University, New York, New York 10032, United States

## Abstract

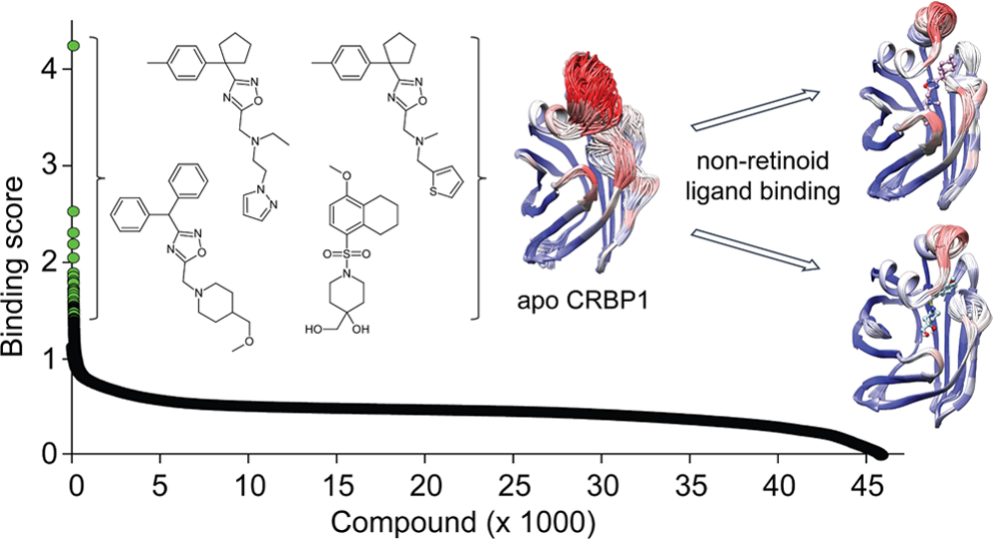

The dysregulation of retinoid metabolism has been linked to prevalent ocular diseases including age-related macular degeneration and Stargardt disease. Modulating retinoid metabolism through pharmacological approaches holds promise for the treatment of these eye diseases. Cellular retinol-binding protein 1 (CRBP1) is the primary transporter of all-*trans*-retinol (atROL) in the eye, and its inhibition has recently been shown to protect mouse retinas from light-induced retinal damage. In this report, we employed high-throughput screening to identify new chemical scaffolds for competitive, nonretinoid inhibitors of CRBP1. To understand the mechanisms of interaction between CRBP1 and these inhibitors, we solved high-resolution X-ray crystal structures of the protein in complex with six selected compounds. By combining protein crystallography with hydrogen/deuterium exchange mass spectrometry, we quantified the conformational changes in CRBP1 caused by different inhibitors and correlated their magnitude with apparent binding affinities. Furthermore, using molecular dynamic simulations, we provided evidence for the functional significance of the “closed” conformation of CRBP1 in retaining ligands within the binding pocket. Collectively, our study outlines the molecular foundations for understanding the mechanism of high-affinity interactions between small molecules and CRBPs, offering a framework for the rational design of improved inhibitors for this class of lipid-binding proteins.

## Introduction

The first step in vision is the activation of visual pigments by light.^1^ These pigments are composed of an opsin apo protein combined with a visual chromophore, 11-*cis*-retinal (11cRAL), and are exclusively expressed in the photoreceptor cells of the retina.^2,3^ The chromophore absorbs a photon of light, inducing photoisomerization of 11-*cis*-retinylidene to its all-*trans* configuration, which activates the visual pigments and triggers the phototransduction signaling pathway.^4,5^ To restore opsins to their light-sensitive state, all-*trans*-retinal (atRAL) needs to be reisomerized to 11cRAL. In vertebrates, this process occurs through a series of enzymatic reactions, collectively known as the visual (retinoid) cycle.^5^ This eye-specific metabolic pathway is crucial for sustainable light perception and the health of photoreceptor cells.^6,7^ Consequently, metabolic deficiencies within the visual cycle can lead to developmental or degenerative retinal disorders.^7,8^

Several mechanisms associated with retinoid metabolism can contribute to retinopathies. For instance, inactivating mutations in *LRAT*, *RDH5*, or *RPE65* genes impairs the production of the visual chromophore and leads to early-onset progressive degeneration of photoreceptors.^9−11^ However, even a functional retinoid cycle can generate cytotoxic metabolites. Certain environmental insults or an unfavorable genetic background can negatively affect ocular retinoid homeostasis and subsequently the retinal function. Despite being essential for vision, atRAL and its metabolites can cause retinal damage, as observed in Stargardt macular dystrophy and age-related macular degeneration (AMD).^12−16^ The cytotoxicity of atRAL is, in part, attributed to the reactivity of its aldehyde group toward certain cellular nucleophiles, including the amino groups of phospholipids and proteins.^12,17,18^ Although the formation of the Schiff base adduct of atRAL with phosphatidylethanolamine is reversible, its reaction with a second molecule of atRAL initiates a cascade of irreversible nonenzymatic conversions. These reactions result in the formation of fluorescent diretinal compounds, including diretinoid-pyridinium-ethanolamine (A2E) and retinaldehyde dimer (RALdi).^19−21^ These compounds sensitize retinal pigment epithelium (RPE) cells to blue-light damage, impair the degradation of phospholipids from phagocytosed rod outer segments, induce the release of proapoptotic proteins from the mitochondria, and destabilize cellular membranes and lysosomes.^22−25^ Consequently, the accumulation of aberrant retinal metabolites, indicated by fundus autofluorescence, precedes macular degeneration and visual loss in Stargardt and AMD patients.

atRAL toxicity and the intracellular deposition of its metabolic side products are prominent features of a malfunctioning visual cycle and aging RPE, contributing to certain retinal diseases. Therefore, finding proper pharmacological targets to regulate the flux of retinoids represents a promising therapeutic approach.^8,26,27^ Multiple binding and transport proteins facilitate retinoid biology, including cellular retinol-binding proteins (CRBPs), with cellular retinol-binding protein 1 (CRBP1) being highly abundant in RPE cells.^28^ CRBP1 enhances intracellular vitamin A uptake and facilitates the recycling of vitamin A from photoreceptor cells.^29−32^ Studies on CRBP1-deficient mice (*Rbp1*^*–/–*^) revealed a diminished amount of all-*trans*-retinyl esters in the RPE and transient accumulation of all-*trans*-retinol (atROL) upon recovery from exposure to bright light.^31^ This phenomenon was accompanied by delayed dark adaptation by a factor of 2 compared to wild-type (WT) mice. Importantly, the deactivation of the *Rbp1* gene does not cause pathological changes in the murine retina.^31^ Additionally, mutations in the RBP1 gene have not been reported to cause human retinal disorders.^33^ Thus, the physiological function of CRBP1 preordains this protein as a pharmacological target for controlling the flux of retinoids in the eye without causing serious ocular side effects.

In our attempt to validate CRBP1 as a pharmacological target, we previously identified abnormal cannabidiol (abn-CBD) as a potent and specific inhibitor of this protein.^34^ We also provided evidence that targeting CRBP1 represents a safe method of controlling retinoid metabolism in the eye.^34^ Here, we report the identification of new chemical scaffolds for competitive inhibitors of CRBP1. We determine binding affinities for the newly identified compounds and elucidate mechanisms of their interaction with the protein by solving high-resolution X-ray structures of human CRBP1 in complex with selected inhibitors. By combining the X-ray crystallography data with hydrogen/deuterium (H/D) exchange mass spectrometry (MS) and molecular dynamics (MD) simulations, we provide detailed mechanistic insight into the structure–function relationship for each of the new classes of CRBP1 inhibitors. Overall, our study outlines the molecular foundations for understanding the mechanism of high-affinity interactions of small molecules with CRBPs and provides a framework for the structure-based design of improved inhibitors for this class of carrier proteins.

## Results

### Identification of Nonretinoid Ligands for CRBP1

**Figure 1:**
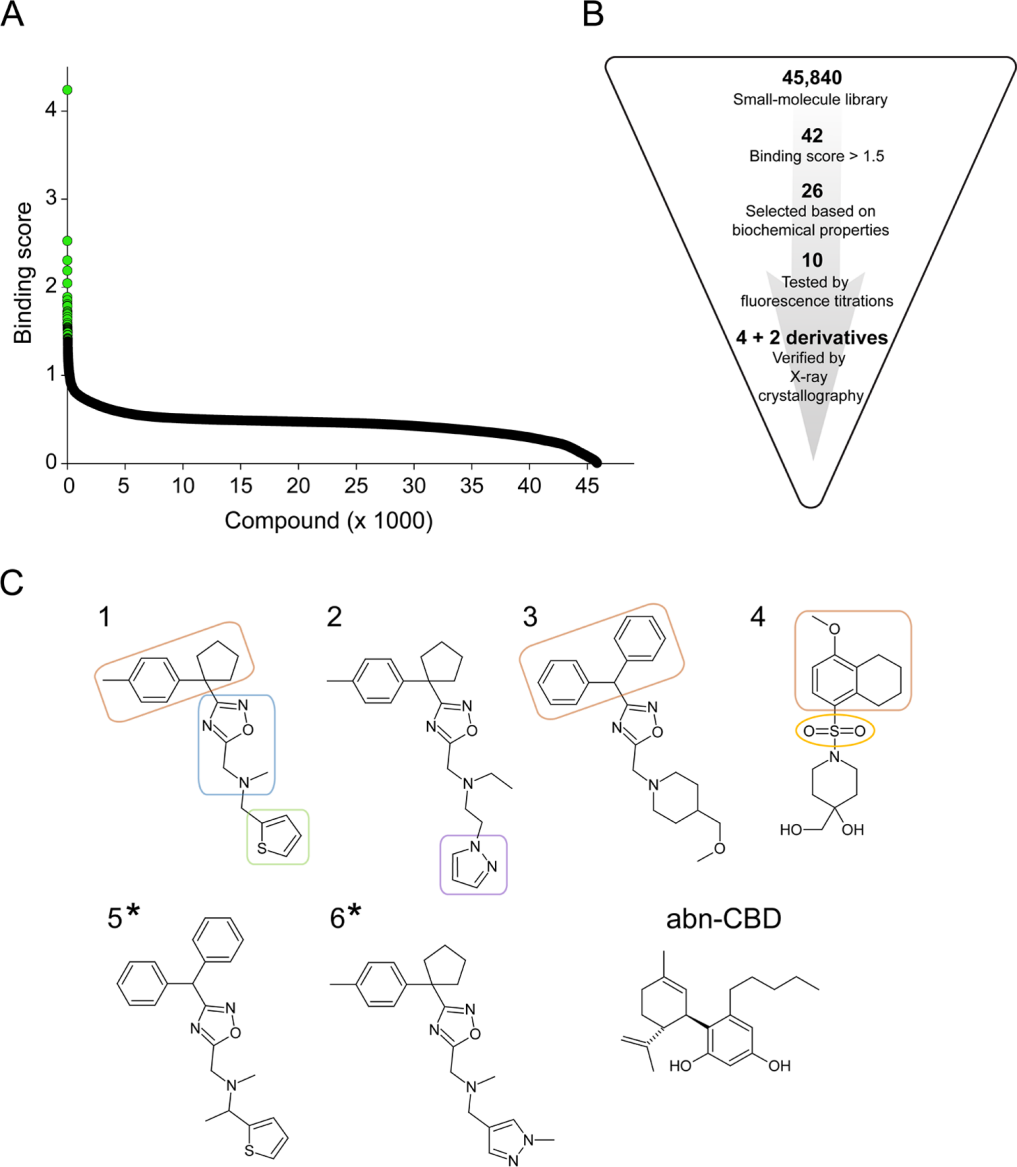
Results of HTS for CRBP1 ligands. (A) Outcome of the HTS of 45,840 compounds from a drug-like small-molecule chemical library. The known high-affinity inhibitor of CRBP1, abn-CBD, was used as a positive control. A mathematical equation in which changes in fluorescence signals at 350 and 480 nm for abn-CBD were used as a reference (see the Materials and Methods section for details) was applied to each measurement in order to distinguish values based on their difference to the positive control. Thus, compounds exhibiting fluorescence changes similar to those observed for abn-CBD received a higher “binding score” compared to chemicals that do not induce fluorescence changes or cause significantly stronger signal than the positive control. Values ≥ 1.5 were considered initial hits (colored green) and further selected based on their spectral and chemical characteristics. (B) Schematic representation of the selection and validation process for the HTS hits. (C) Chemical structure of six compounds (numbered 1–6) that were confirmed to be present in the binding pocket of CRBP1 by X-ray crystallography. Compounds 1–4 were identified directly from HTS, whereas compounds 5 and 6 (denoted with asterisks) were tested derivatives found based on the subsequent structure/function analysis (Figure S2). The chemical structure of abn-CBD was added for reference. The compounds can be divided into two structurally distinct subgroups represented by inhibitors 1–3 and inhibitor 4. The common structural element of the newly identified CRBP1 inhibitors is a 1,2,4-oxadiazol ring (blue) linked via a tertiary amine to a thiophene (green) or pyrazol ring (violet). A bulky hydrophobic moiety (brown) is represented by 4-methylphenyl cyclopentyl (inhibitors 1 and 2) or diphenylmethyl moieties (inhibitor 3). A sulfonyl group (marked yellow) distinguishes inhibitor 4 among the HTS hits. It is linked to a hydrophobic methoxy-tetrahydronaphthalene moiety and a piperidine ring derivative, also present in inhibitor 3.

abn-CBD was previously identified as an inhibitor of CRBP1 by screening a relatively small (∼1000 compounds) library of selected lipid compounds (Cayman Chemicals and Enzo Life Science).^34^ An obvious limitation of this initial approach was the narrow variety of tested chemical scaffolds. A more diverse and drug-like library of chemicals would be advantageous for identifying alternative small-molecule inhibitors of CRBP1 with comparable or superior pharmacodynamic properties. Also, the pharmacokinetic characteristic of abn-CBD, especially for oral drug administration, is not favorable. Hence, one of the primary objectives of our research was to discover alternative inhibitors with potentially improved bioavailability and longer half-life compared to abn-CBD. Hence, in our comprehensive endeavor to thoroughly investigate the chemical space for small-molecule inhibitors of CRBP1, we harnessed a substantially expanded library consisting of 45,840 small-molecule compounds characterized by drug-like properties (obtained from ChemBridge). Given the well-established high-binding affinity of abn-CBD to CRBP1, this particular compound was strategically employed as a reference within the framework of the high-throughput screen (HTS) assay^34^ (Figure 1).

The initial pool of hit compounds was scrutinized for exclusion criteria such as UV/vis absorbance or autofluorescence that could have interfered with the HTS assay (Figure 1B). Subsequent validation of the preselected HTS hits involved the utilization of a CRBP1 crystallization assay. This assay capitalizes on the distinction that holo CRBP1, unlike the apo form, readily forms well-defined macromolecule crystals. Employing crystallization as a secondary screening method aligns with the rigorous standards anticipated for the definitive identification of new inhibitors. Thus, hit compounds that demonstrated binding affinity through fluorescence titrations were excluded from consideration if they failed to cocrystallize with CRBP1 due to potential nonspecific binding or perturbation of the fluorescence assay. The list of such compounds is detailed in Figure S1. This approach resulted in the final selection of four compounds (Figure 1B,C and Table 1).

**Table 1:** Inhibitors of CRBP1 identified by HTS and their *K*_i_ values

inh. no.	PubChem ID	Hit2Lead ID	IUPAC name	PDB ligand name (ligand code)	log *P*^a^	*K*_*i*_ (μM)
1	25370031	28421637	*N*-methyl-*N*-[[3-[1-(4-methylphenyl)cyclopentyl]-1,2,4-oxadiazol-5-yl]methyl]-1-thiophen-2-ylmethanamine	*N*-methyl-1-{3-[1-(4-methylphenyl)cyclopentyl]-1,2,4-oxadiazol-5-yl}-*N*-(2-thienylmethyl)methanamine (Z5H)	4.9	9.0 ± 4.1
2	42194644	73251475	*N*-ethyl-*N*-[[3-[1-(4-methylphenyl)cyclopentyl]-1,2,4-oxadiazol-5-yl]methyl]-2-pyrazol-1-ylethanamine	*N*-ethyl-*N*-({3-[1-(4-methylphenyl)cyclopentyl]-1,2,4-oxadiazol-5-yl}methyl)-2-(1H-pyrazol-1-yl)ethan-1-amine (ZDF)	4.0	7.1 ± 2.7
3	42094040	10035007	3-benzhydryl-5-[[4-(methoxymethyl)piperidin-1-yl]methyl]-1,2,4-oxadiazole	1-{[3-(diphenylmethyl)-1,2,4-oxadiazol-5-yl]methyl}-4-(methoxymethyl) piperidine (ZDK)	4.0	8.3 ± 2.8
4	56739971	17484185	4-(hydroxymethyl)-1-[(4-methoxy-5,6,7,8-tetrahydronaphthalen-1-yl)sulfonyl]piperidin-4-ol	4-(hydroxymethyl)-1-(4-methoxy-5,6,7,8-tetrahydronaphthalene-1-sulfonyl) piperidin-4-ol (ZE2)	1.4	9.5 ± 3.7
5	45209226	42757275	*N*-[(3-benzhydryl-1,2,4-oxadiazol-5-yl)methyl]-*N*-methyl-1-thiophen-2-ylethanamine	(1S)-*N*-{[3-(diphenylmethyl)-1,2,4-oxadiazol-5-yl]methyl}-*N*-methyl-1- (thiophen-2-yl)ethan-1-amine (ZA6)	5.0	10.6 ± 2.5
6	16188253	33562185	*N*-methyl-*N*-[[3-[1-(4-methylphenyl)cyclopentyl]-1,2,4-oxadiazol-5-yl]methyl]-1-(1-methylpyrazol-4-yl)methanamine	*N*-methyl-1-{3-[1-(4-methylphenyl)cyclopentyl]-1,2,4-oxadiazol-5-yl}-*N*-[(1-methyl-1H-pyrazol-4-yl)methyl]methanamine (ZCF)	3.5	10.7 ± 4.5

a log *P* values as provided by PubChem.

Three of the newly identified inhibitors of CRBP1 are structurally related by containing a 1,2,4-oxadiazol ring (inhibitors 1–3). The common feature of these compounds is the substitution of the oxadiazol ring at position 3 with a bulky moiety such as 4-methylphenyl cyclopentyl (inhibitors 1 and 2) or diphenylmethyl (inhibitor 3). Position 5 of the oxadiazole ring is coupled to either a thiophene ring (inhibitors 1), a pyrazole ring (inhibitor 2), or a methoxymethylpiperidine moiety present in inhibitor 3. A distinct structural motif is observed in inhibitor 4, which is a derivative of methoxytetrahydronaphthalenesulfonate substituted with a 4-hydroxymethylpiperidine moiety (Figure 1C).

As part of the structure/function analysis of the principal chemical scaffolds, closely related derivatives of inhibitors 1–4 were tested. As depicted in Figure S2A, substitution of the hydrophobic thiophene ring in inhibitor 1 with a polar methyl pyrazole (inhibitor 6) had no impact on the binding to CRBP1. However, altering the tertiary amine with a dimethyl imidazole ring resulted in an undetectable binding affinity via fluorescence titration and inability to cocrystallize with CRBP1. Replacing the diphenylmethyl moiety in inhibitor 3 with the smaller and more polar trifluoromethyl group abolished the interaction with CRBP1, underscoring the significance of hydrophobic interactions between a ligand and the portal region, similar to those observed for atROL (Figure S2B). As anticipated, the substitution of the hydrophobic 4-methylphenyl cyclopentyl group in inhibitor 1 with diphenylmethyl moieties yielded a functional CRBP1 inhibitor, denoted as compound number 5. We also explored whether a sulfonyl group could play a central role in the binding process. To this end, we examined a compound structurally similar to inhibitors 1–3, but featuring a sulfonyl group instead of the oxadiazol ring and a methyl group in lieu of the cyclopentyl moiety (Figure S2C). However, such a modified compound did not demonstrate any evident binding to CRBP1. Although partial, these analyses clearly outlined the structural parameters that molecules must adhere to in order to maintain stable associations with CRBP1.

### Determination of the Binding Affinities for CRBP1 Inhibitors

**Figure 2:**
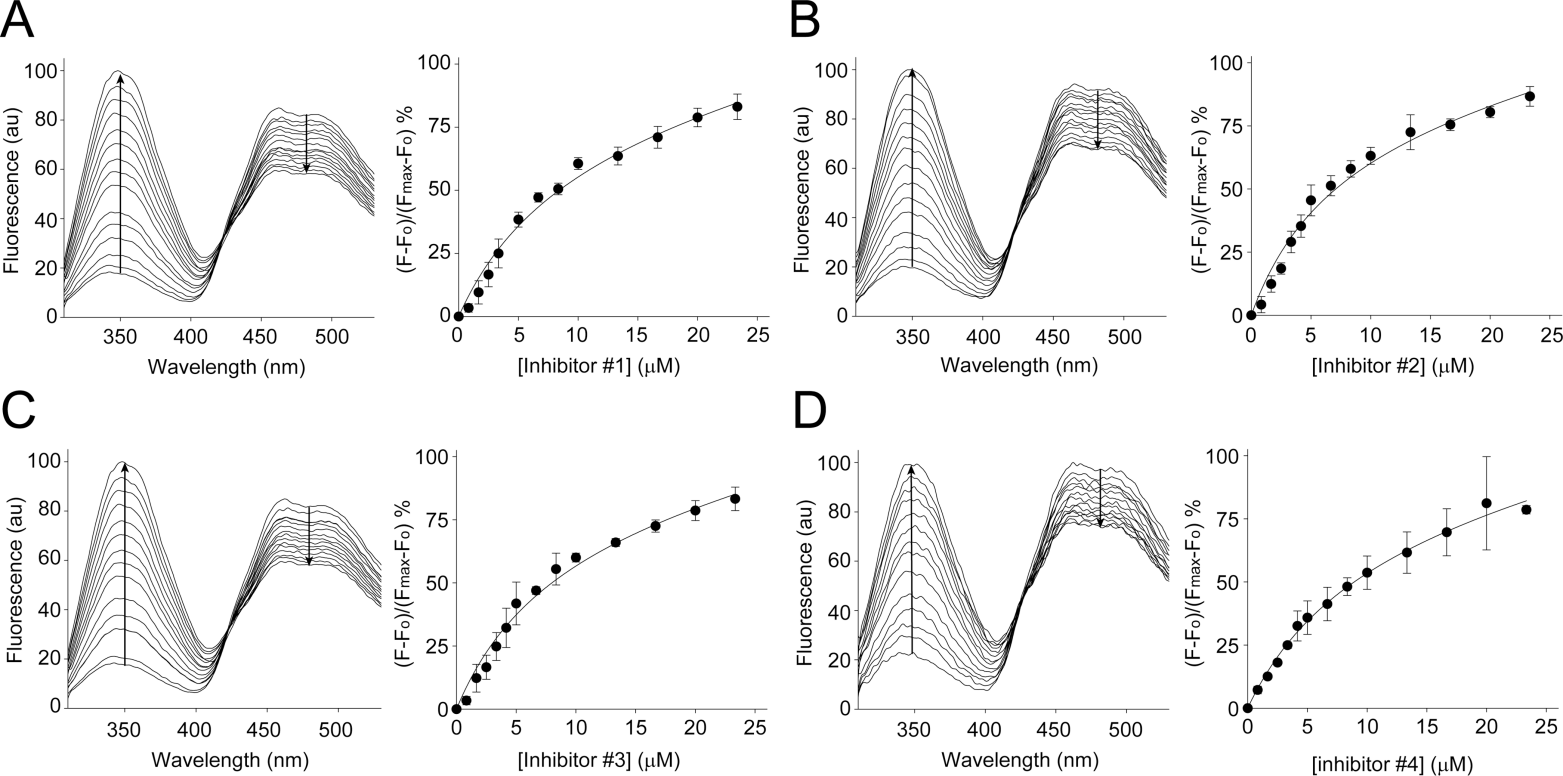
Determination of *K*_i_ values for CRBP1 inhibitors. The interaction of the selected CRBP1 inhibitors with the protein was quantified using a fluorescence assay in which a tested compound outcompetes atROL bound to the protein. Panel (A) represents the titration with increasing concentrations of inhibiotr 1, panel (B) corresponds to inhibitor 2, whereas panels (C) and (D) show data for inhibitors 3 and 4, respectively. Arrows indicate direction of changes in the fluorescence signal upon titration. Emission values at 350 nm were plotted against inhibitor concentration and fitted with a one-site binding saturation model with a nonspecific binding component. The calculated *K*_i_ values for all examined inhibitors are given in Table 1. The error bars correspond to standard deviation values calculated for three independent titrations.

To assess the potency of the interaction of CRBP1 with the newly identified inhibitors, we determined the *K*_i_ values for each of the compounds using an atROL-replacement assay. For this purpose, the CRBP1/atROL complex was incubated with increasing concentrations of the tested inhibitors. The liberation of atROL from the binding pocket of CRBP1 in the presence of selected nonretinoid compounds was monitored by the disruption of the fluorescence resonance energy transfer (FRET) between the protein scaffold and the retinoid moiety, resulting in an increase in protein fluorescence at 350 nm and a decrease at 480 nm (Figure 2).^34^

Changes in the fluorescence signal were plotted as a function of inhibitor concentration and fitted with a one-site saturation-binding equation with a nonspecific binding component. The calculated values of *K*_i_ for the examined compounds were around 10 μM (Table 1). Notably, the affinities of the newly identified inhibitors for CRBP1 were lower with *K*_i_ values of 7.1–10.7 μM compared to abn-CBD (*K*_i_ value of 67 nM).^34^

### Structural Basis for the Interaction of CRBP1 with Its Inhibitors

**Figure 3:**
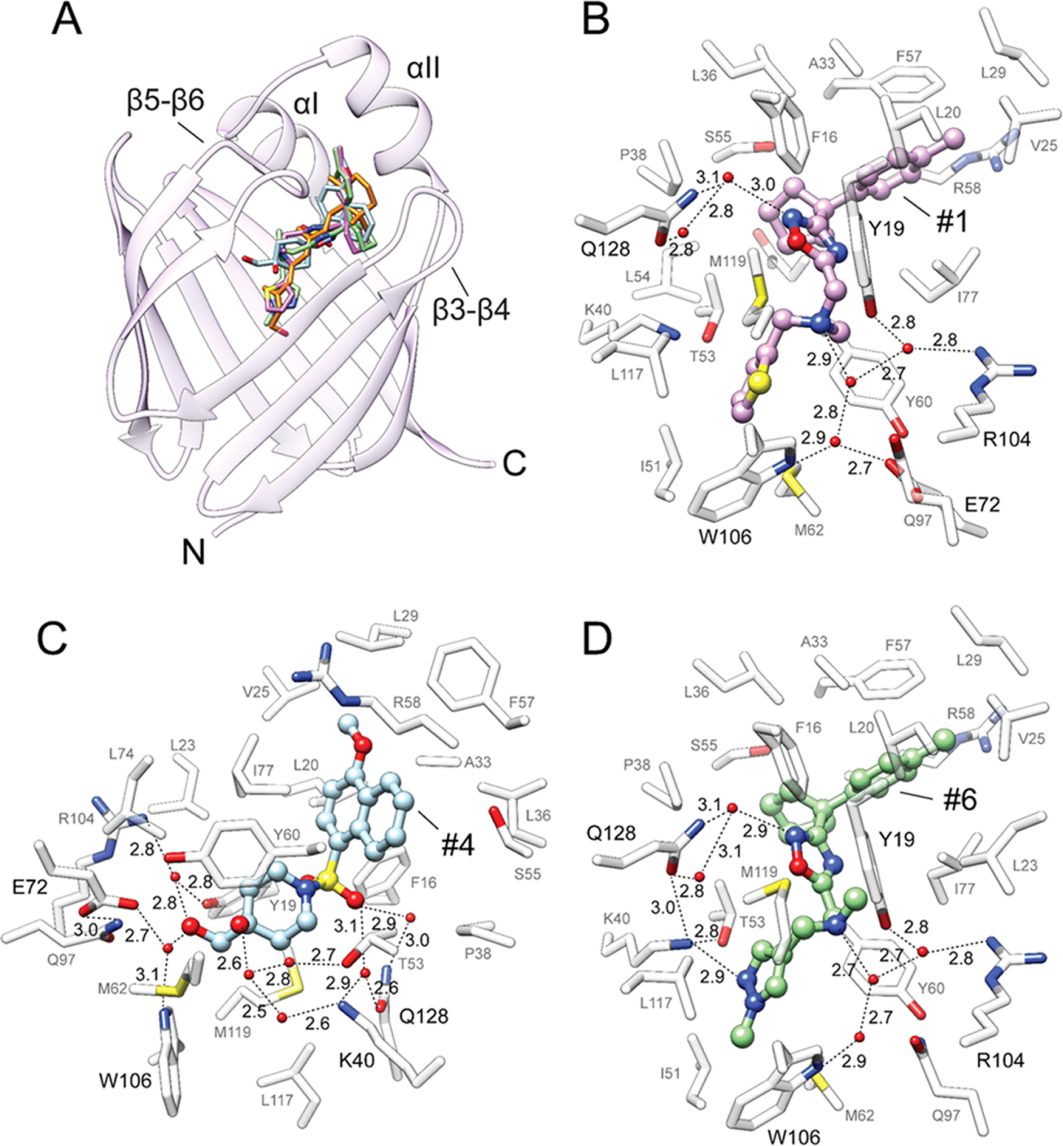
Crystal structure of CRBP1 in complex with its inhibitors. (A) Overlay of the positions of atROL (orange, PDB 5H8T), inhibitor 1 (purple, PDB 8GD2), inhibitor 4 (blue, PDB 8GEY), and inhibitor 6 (green, PDB 8GEU). α-helices I/II and β-hairpins 3–4 and 5–6 constitute the conformationally flexible portal region of the protein, which covers the entrance to the binding pocket. (B–D) Molecular details of the interactions of inhibitors 1 (B), 4 (C), and 6 (D) within the binding pocket of CRBP1. Ordered water molecules are shown as red spheres; dashed lines indicate hydrogen bonds. Distances are shown in angstroms.

To confirm the binding and understand the mode of interaction, we crystallized CRBP1 in complex with the identified compounds. The high resolution of the X-ray diffraction data, ranging between 1.13 and 1.85 Å (Table S1), allowed for unambiguous identification of the electron density of the ligands, enabling accurate modeling of these compounds in the binding pocket (Figure S3). Consistent with the fluorescence data, all of the crystallized compounds acted as competitive inhibitors of CRBP1, occupying the same binding site as atROL (Figures 3A and S3). However, the mode of interaction within this site differed considerably between atROL and the nonretinoid ligands. The binding of the planar and rigid atROL primarily occurs through hydrophobic and van der Waals interactions between the β-ionone ring and the polyene chain of the retinoid moiety with the nonpolar side chains inside the ligand-binding cavity.^35,36^ The interaction with CRBP1 is further stabilized by a hydrogen bond between the hydroxyl group of atROL and the side chains of Q108 and K40.^35^

Although the overall position of the nonretinoid ligands within the CRBP1 binding site essentially overlaps with atROL, the presence of several potential hydrogen bond acceptors allows them to interact with side chains that are not typically involved in the interaction with retinoids. For example, compounds that contain the 1,2,4-oxadiazole ring form a hydrogen bond between their nitrogen atom N(2) and an ordered water molecule, whose spatial position is further stabilized by the interaction with the side chain of Q128 (inhibitors 1, 3, 5, and 6) (Figures 3B,D and S4B,C). In inhibitor 2, an alternative hydrogen bond is observed between the oxygen atom of the oxadiazole ring and two ordered water molecules (Figure S4A). A secondary interaction site is formed between the nitrogen atom of the *N*-methyl group and the ordered water molecule (inhibitor 1, 5, and 6), which in turn is involved in an extended network of hydrogen bonding with the side chains of residues Y19, W106, and R104 (Figures 3B,D and S4C). Similar interactions are preserved in inhibitor 3, where the *N*-methyl group is replaced by a piperidine moiety (Figure S4B). Interestingly, the presence of an N-ethyl group in inhibitor 2, induces an alternative conformation of this molecule in the binding site, which no longer supports hydrogen bonding of the tertiary amine group (Figure S4A). Another characteristic interaction site is observed for inhibitors 2 and 6, involving the N(2) nitrogen of their pyrazole rings. In ligand 2, a hydrogen bond is formed between N(2) and an ordered water molecule, which is further stabilized by its interactions with the side chain of T53 (Figure S4A). In contrast, the 1-methyl pyrazole moiety in inhibitor 6 is involved in an extended network of hydrogen bonds, including the side chains of K40, Q128, and T53, as well as adjacent ordered water molecules (Figure 3D). In inhibitor 3, the pyrazole ring is substituted with a methoxymethyl moiety. Nevertheless, hydrogen bond interactions are preserved as oxygen in the vicinity (3.0 Å) of the ζ-nitrogen atom of K40 (Figure S4B).

Inhibitor 4 stands out from the other compounds identified in the HTS due to its distinct structure (Figure 1C). It contains a sulfonyl group and two hydroxyl groups, which provide alternative sites for interactions with the protein scaffold. The oxygen atom of the hydroxymethyl group participates in an extended network of hydrogen bonds involving two ordered water molecules and the side chains of E72, W106, Y19, and R104 (Figure 3C). The second hydroxyl group of inhibotor 4 is also engaged in a hydrogen bond network with several crystallographic water molecules. Additionally, one of the oxygen atoms of the sulfonyl group faces a polar patch within the binding pocket, creating an environment suitable for the formation of an additional hydrogen bond. Specifically, the oxygen atom indirectly interacts with the side chains of Q128 and K40 through two ordered water molecules situated between the sulfonyl group and these residues (Figure 3C).

Apart from specific hydrogen bonds, effective binding of retinoids or other high-affinity ligands to CRBPs requires interactions with the entry portal region of the protein, comprising α-helix I and II, as well as hairpin turns between β-strands 3–4 and 5–6 (Figures 3A and S5A).^34,35,37^ This segment undergoes reduced flexibility upon interaction with specific ligands, stabilizing the “locked” conformation of the proteins and decreasing the *k*_off_ rate of the bound compound.^38−40^ The binding of atROL induces a conformational change in this region through hydrophobic and van der Waals interactions of the β-ionone ring with a nonpolar cleft formed by the portal region.^35^ Similar interactions are observed for each of the newly discovered inhibitors. The spatial position of these compounds reveals an overlap between the orientation of the β-ionone ring of atROL and the methylphenyl or cyclopentyl rings (inhibitors 1, 2, and 6), diphenylmethyl rings (inhibitors 3 and 5), or the tetrahydronaphthalene moiety of inhibitor 4 (Figures 3A and S5A). Thus, the bulky, cyclic, and hydrophobic structural elements present in these compounds appear to be crucial motifs that, in combination with specific polar interactions enable binding to CRBP1.

To assess whether the newly identified inhibitors demonstrate specificity for particular members of the CRBP protein family, we conducted a compared the binding site architecture of human CRBP1 with CRBP2, CRBP3, and CRBP4 (Figure S6). Our analysis revealed the absence of amino acid substitutions that could directly hinder the binding of inhibitors 1–6 to these closely related proteins. Moreover, the critical amino acids involved in polar interactions with these compounds remain conserved across all human CRBPs. As a result, we infer that unlike abn-CBD,^34^ the investigated inhibitors lack specificity for individual CRBPs.

### Changes in the Protein Structure Induced by Ligand Binding

atROL and all of the identified nonretinoid inhibitors of CRBP1 utilize the same binding site. However, the nonretinoid compounds are engaged in numerous specific hydrogen bonds that are not possible with the retinoid moiety. Despite these enhanced interactions, the binding affinity of atROL is much higher than those of the identified inhibitors (Table 1). Only, the previously identified abn-CBD demonstrates a binding affinity comparable to that of atROL.^34^ To investigate the structural factors that govern the overall affinities of protein–ligand interactions, the binding models of selected compounds were correlated with their impact on the conformational flexibility of CRBP1. When comparing the spatial position of inhibitors 1–6 and abn-CBD (high-affinity ligand), it is observed that the hydrophobic ring of these molecules overlaps with the cyclohexene ring of abn-CBD (Figure S5B). However, the benzenediol ring of abn-CBD occupies a space in the binding pocket that is not utilized by any other identified inhibitor or atROL. Moreover, the hydroxyl group of the benzenediol ring in the para position participates in a hydrogen bond network involving the side chains of Q128, while the ortho hydroxyl interacts with the carboxyl oxygen of the A33 residue (Figure S5B). A33 is located within α-helix II, which is an integral part of the portal region. Hence, the interaction with the main chain of A33 appears to be particularly important for the stabilization of the closed conformation of CRBP1 upon binding of abn-CBD. Noticeably, none of the other examined compounds form polar interactions with the portal region of the protein. Based on this observation, we hypothesized that hydrogen bonds with amino acids of the portal region constitute an alternative mechanism through which a nonretinoid ligand can stabilize a tightly closed conformation of CRBP1.

**Figure 4:**
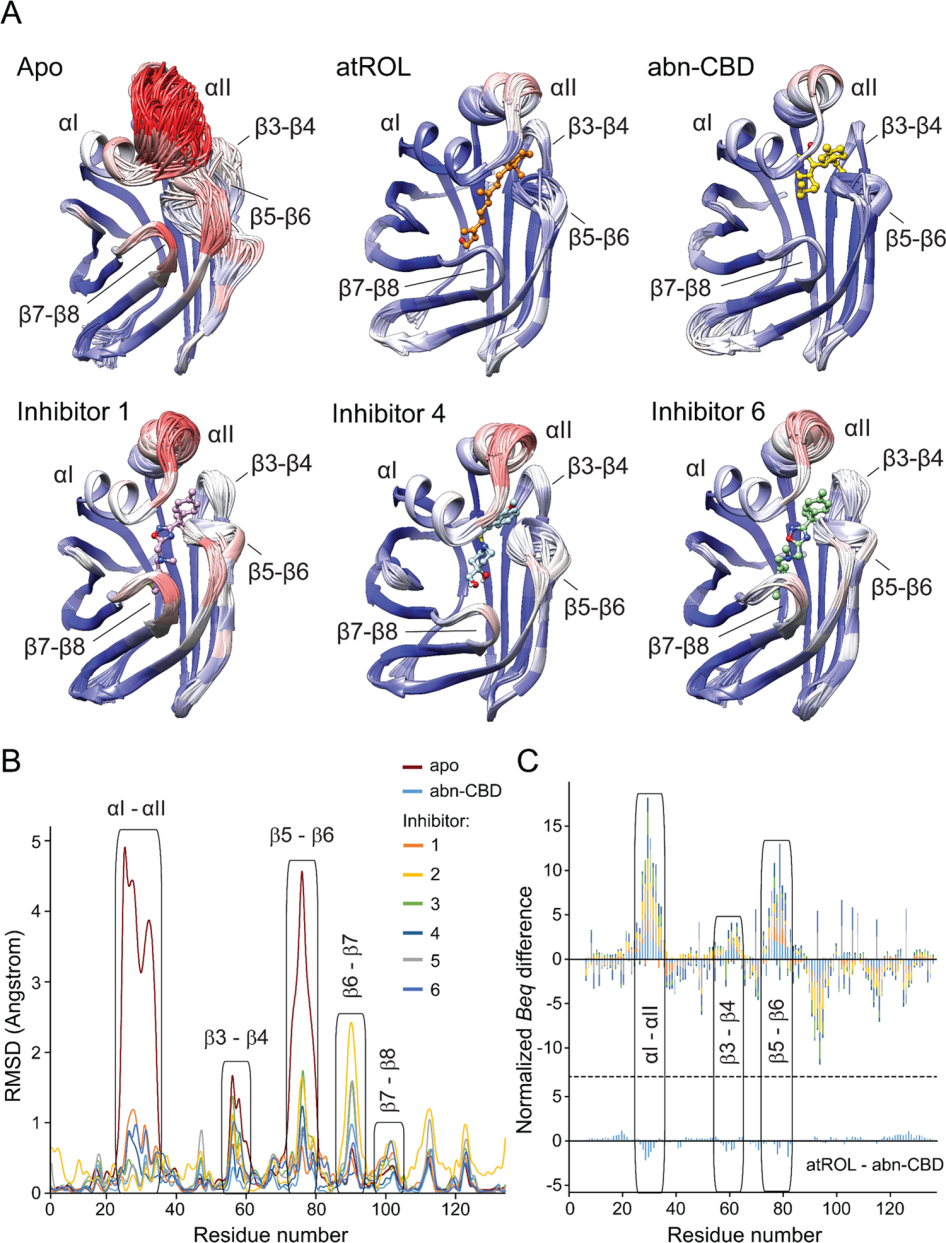
Changes in the structural dynamics of CRBP1 upon interaction with atROL and nonretinoid inhibitors. (A) Cartoon representation of the ensemble refinement of the crystallographic structures of CRBP1 in the apo and ligand-bound states. The color scheme represents the average *B*-factors per residue, with the highest values marked in red and the lowest values in blue. Superimposition of individual structures of the assemblies revealed high flexibility of the portal region in the apo protein, specifically α-helix II and the loop between β-strands 5 and 6. (B) Quantification of differences in the positions of individual CRBP1 structures resulting from the crystallographic ensemble refinement. RMSD differences were calculated for the main chain of each residue using Chimera software version 1.16. (C) Comparison of the differences in normalized equivalent *B*-factors for apo and ligand-bound CRBP1 structures. The *B*_eq_ values for the main chain of each residue in the holo structures were subtracted from the corresponding *B*_eq_ values of the apo protein. The graph shows the cumulative differences and the contribution of individual structures of CRBP1 bound to inhibitors 1–6 and abn-CBD. The protein regions with increased conformational dynamics are labeled. The color scheme corresponds to panel (B). For comparison, the differences in the *B*_eq_ values for the protein structures in complex with atROL and abn-CBD are shown at the bottom of the panel.

To verify this assumption, we employed crystallographic ensemble refinement to explore the conformational flexibility of CRBP1 bound to different ligands.^41^ This method allows for sampling local molecular vibrations of X-ray models and combining them with MD simulations to visualize the fluctuations of atoms with multiple structural states. Importantly, all of the examined CRBP1 crystals were isomorphous, minimizing the risk of introducing artifacts related to variations in the crystal packing. As expected, comparison of the CRBP1/atROL complex to the apo form revealed high flexibility of the portal region in the absence of a ligand, as evidenced by a wide range of alternative conformations (Figure 4A). Importantly, the relatively flexible section of the portal region (α-helices I/II) experienced partial stabilization through the presence of compounds 1–6. This is evident from their structural ensembles, which exhibit greater similarity to CRBP1 in complex with atROL or abn-CBD than the apo form (Figures 4 and S7). Since the interaction with abn-CBD closely emulates the dynamic behavior of the protein scaffold as observed with the natural ligand, discernible variations arise in the ability of inhibitors 1–6 to foster a closed conformation within the portal region.

To quantify the differences in structural stability, we computed the RMSD for each residue within complexes of CRBP1 with its ligands. As illustrated in Figure 4B, the stability of the main chain in the α-helices I/II and the loop between β-strands 5 and 6 correlates with the binding affinity of the tested compounds, showing a notable reduction for ligands with lower binding affinities. It is noteworthy that inhibitor 2, 3, and 5 exhibit a greater propensity to stabilize the α-helices I/II region in comparison to compounds 1, 4, and 6. Additionally, it is interesting to note that certain nonretinoid ligands have the potential to enhance conformational flexibility in segments of CRBP1 that remain well-structured even in the protein’s apo form. This phenomenon is particularly observed in inhibitors 2, 3, and 5, where their binding leads to a partial destabilization of β-strands 6 and 7 (Figures 4B and S7).

Furthermore, we compared the isotropic crystallographic *B*-factors for the main chain of CRBP1 bound to atROL, abn-CBD, and newly discovered inhibitors. To account for refinement strategies and X-ray data resolution differences, the *B*-factor values were scaled through z-score normalization to calculate equivalent isotropic crystallographic *B*-factors (*B*_eq_) before the analysis.^42^ As a control, we calculated the corresponding differences in *B*_eq_ for the CRBP1 structures in complexes with atROL and abn-CBD. Analysis of the normalized crystallographic *B*-factors between the apo and holo forms of CRBP1 indicates regions of reduced backbone mobility, which are conserved and localized at the portal region for all examined compounds (Figure 4C). Consequently, ligand binding resulted in decreased relative *B*-factor values, suggesting reduced conformational flexibility in this region of CRBP1. The most significant differences between the apo and holo forms of the protein were observed in α-helix II and the loop between β-strands 5–6, as exemplified by the RMSD values (Figure 4B). Interestingly, the magnitude of changes in conformational flexibility depends on the type of inhibitor. abn-CBD and inhibitors 2, 3, and 5 exhibited the strongest stabilizing effect on the portal region, followed by inhibitors 1, 4, and 6 (Figure 4B,C). Therefore, the ability to preserve the closed conformation of the protein is a characteristic feature of compounds that bind the protein with nanomolar affinity, such as atROL and abn-CBD. In contrast, inhibitors with *K*_i_ values in the micromolar range allow for partial flexibility of the portal region.

### Ligand-Induced Alterations in the Conformational Dynamics of CRBP1

**Figure 5:**
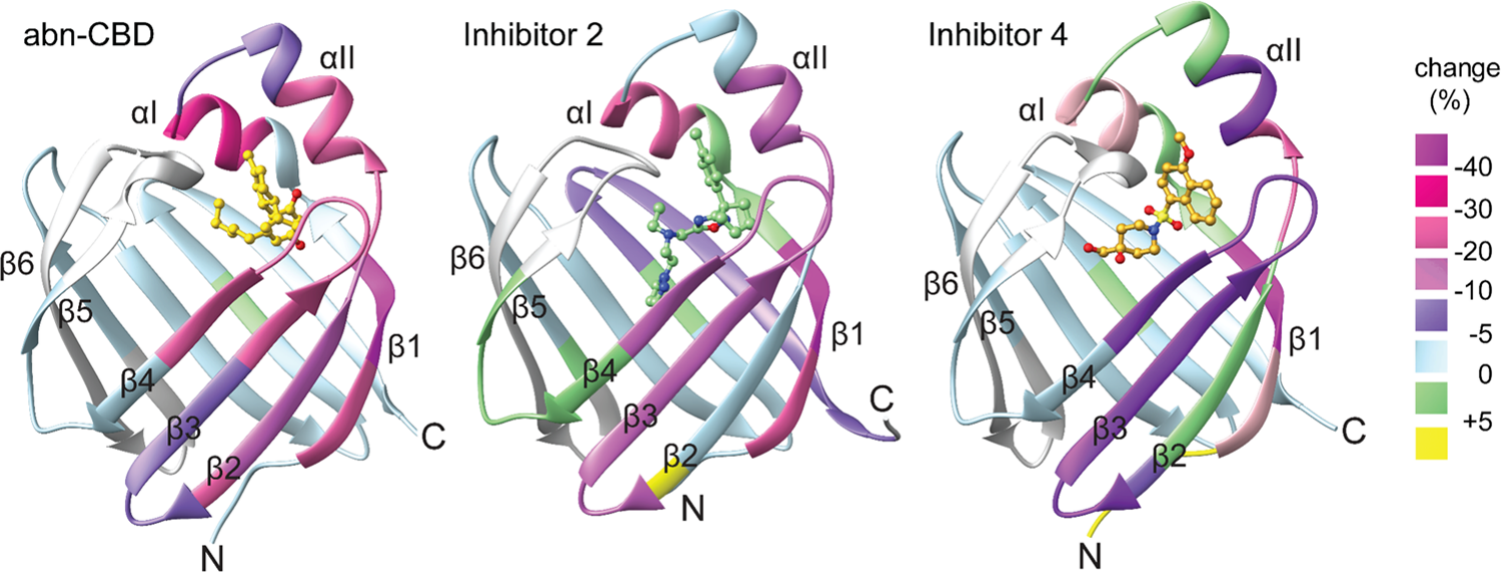
H/D exchange differential map of apo and inhibitor-bound CRBP1. The percentage of deuteration for the inhibitor-bound state was subtracted from that of the apo form and displayed on the tertiary structure of the protein. Negative differences in percent deuteration indicate a more stable secondary structure and less conformational flexibility in particular regions (from purple to pink). The changes in deuterium uptake between abn-CBD and inhibitors 2 and 4 indicate increased conformational flexibility of the protein scaffold upon binding of the latter compounds.

It is important to note that the dynamics of proteins in the crystal lattice may not fully replicate the conformational flexibility observed in solution due to intermolecular contacts. To verify and compare the conformational flexibility of CRBP1 in solution, we conducted H/D exchange experiments. The H/D exchange experiments provide insights into the conformational dynamics of the protein by measuring the efficiency of deuterium uptake. Consistent with previous studies,^43,44^ we observed increased deuterium uptake, indicating conformational flexibility in the apo CRBP1 (Figures 5 and S8). Notably, regions with a particularly high H/D exchange were identified, primarily within the portal region, including α-helices I/II and portions of the β-strand 3/4 hairpin. Additionally, β-strand 1 exhibited a relatively high deuterium uptake. By comparing the H/D exchange efficiency between the apo- and abn-CBD-bound forms of CRBP1, we observed significant changes in deuterium uptake.

A dramatic reduction in the level of deuterium uptake was evident in the portal region of both atROL- and abn-CBD-bound CRBP1. The most pronounced difference was observed in α-helix I and II, which form a cap over the entrance to the binding site (Figures 5 and S8). The stabilization of the secondary structure in this region by abn-CBD is likely attributed to direct interactions between the ligand and the main chain of α-helix II (A33). A similar decrease in H/D exchange was observed in β-strand 1, which also makes direct contact with the abn-CBD molecule through interaction with the side chain of Q128.^34^ Notably, a decline in deuterium uptake was also observed when CRBP1 interacted with the newly identified compounds, represented here by inhibitors 2 and 4 (Figures 5 and S8). The reduced H/D exchange for the tested compounds was observed in the same region of the protein as for abn-CBD. However, the level of deuterium uptake was higher compared to the high-affinity inhibitor, indicating increased conformational flexibility of the portal region. Consequently, a more relaxed backbone in this critical region of the protein leads to less stable holo CRBP1 complexes. In summary, compounds that fail to sufficiently stabilize the “closed” conformation of CRBP1 through preferential interactions with α-helix I or II cannot effectively interact with the protein. The higher *k*_off_ rates of these inhibitors likely contribute to their lower affinities compared to abn-CBD.

**Figure 6:**
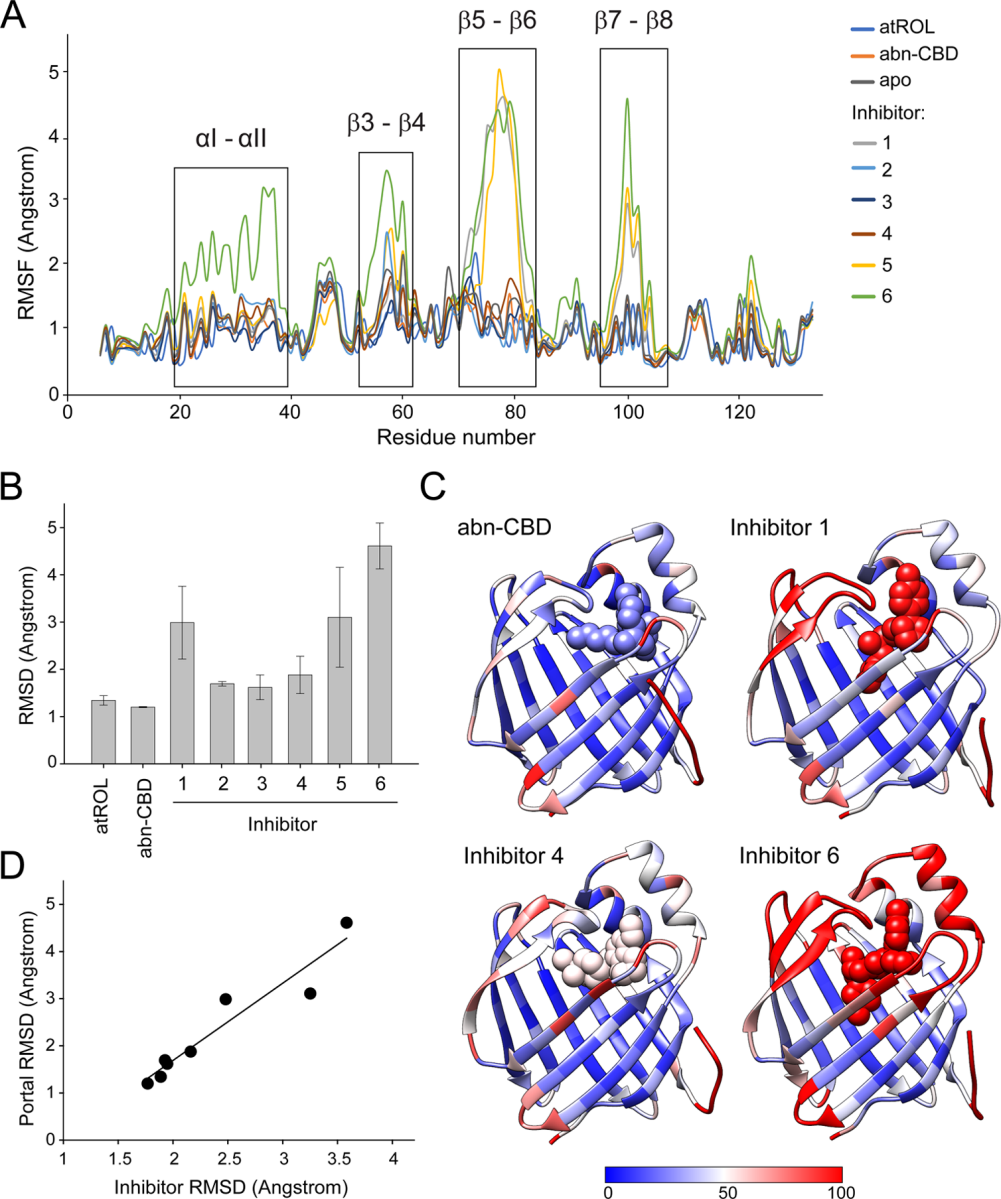
Molecular dynamics simulations of apo and holo CRBP1. (A) Average per-residue RMSF values for all nine CRBP1 systems. Four high-dynamic regions are highlighted: αI–αII loop, β3–β4 loop, β5–β6 loop, and the β7–β8 loop. Average RMSF values were calculated from duplicate simulations. (B) Average RMSD value for each of the eight ligands tested. The values shown as average of both replicates ± SD. (C) Average per-residue *B*-factors of four different ligand-bound systems: abn-CBD and inhibitors 4–6. Simulated *B*-factors were computed by using the atomic fluctuations in cpptraj. The values were obtained by squaring atomic positional fluctuations and weighting these values by 8/3 π^2^. Color scale ranges from blue (*B*-factor of 0), representing stable residues, to red (*B*-factor of 100), which represents dynamic regions. (D) Correlation of the average RMSD value of the ligands bound to CRBP1 to the variability in the conformation of the portal region (αI–αII and loops between β3–β4, β5–β6). Data are correlated with an *R*^2^ value of 0.91.

To add another component to the understanding of CRBP1 dynamics as observed in the H/D exchange, MD simulations on nine different CRBP1 systems (apo CRBP1 and holo CRBP1 bound to atROL, abn-CBD, and inhibitors 1–6) were performed. All MD simulations were performed for 500 ns in duplicate, resulting in 9 μs of total simulation time (Table S2). Trajectories were aligned using the β-strands of CRBP1 and per-residue root-mean-square fluctuation (RMSF) values were calculated to visualize protein flexibility. Additionally, heavy-atom RMSD values were established to characterize the stability of the ligand in the binding pocket and assess the portal region dynamics, focusing on the αI–αII, β3–β4, and β5–β6 loops. The results confirm that the portal region displays different degrees of flexibility in both apo and holo CRBP1 systems (Figure 6). This is highlighted by per-residue RMSF analysis, which shows that the four areas in the portal region (αI–αII and loops β3–β4, β5–β6 and β7–β8) vary in flexibility over the nine systems simulated (Figure 6A).

The most notable is the β5–β6 loop, where the binding of the high-affinity ligands atROL and abn-CBD highly stabilizes this region, with RMSD values of 1.53 and 1.69 Å, respectively. Meanwhile, the newly identified inhibitors 1–6 allow for greater flexibility of this part of the protein (RMSD values ranging from 1.74 to 5.22 Å) (Figure S9A and Table S2). The high-affinity ligands atROL and abn-CBD, which effectively stabilize the portal region, remain tightly bound in the pocket throughout the simulations shown by RMSD values of 1.34 and 1.20 Å, respectively. In comparison, the inhibitors identified through HTS, with significantly weaker binding affinities, exhibit more flexibility in the pocket, with RMSD values ranging from 1.62 to 4.61 Å (Figure 6B).

In summary, a pattern emerges in which overall ligand stability and affinity are associated with the closing and stabilization of the portal region. This is supported by *B*-factor analysis of the MD simulations, which demonstrate that increased ligand dynamics are coupled with increased motion in the portal region, while the β-sheets and posterior loops remain relatively stable during the simulations (Figure 6C). Moreover, averaged portal region RMSD values correlate with the experimentally determined *K*_i_ values for the six tested inhibitors, with an *R*^2^ value of 0.78, where increased motion predicts a weaker binder (Figure S9B). This increased motion most likely leads to more exchange and higher off rates for the weaker ligands. In fact, decreased stabilization of the portal region results in flexibility of the CRBP1 binding pocket and, consequently, lower binding affinity, as illustrated by a strong correlation between RMSD values for the ligands and the portal region (Figure 6D). These findings align well with the crystallographic *B*-factor and H/D exchange experiments, where weaker-affinity ligands were shown to allow for more conformational flexibility in this region (Figures 4 and 5).

## Discussion

Modulation of the metabolic flow of retinoids through the visual cycle is a promising concept for therapies against retinal degenerative diseases associated with the overaccumulation of side products of retinal reactivity. However, previous pharmacological strategies targeting retinoid isomerization of RPE65 were unsuccessful, mostly due to the inability to achieve a balance between beneficial and adverse effects of therapies.^45^ To minimize the side effects, such as night blindness and dyschromatopsia, associated with the direct blockage of 11cRAL production by RPE65 inhibitors, alternative strategies were developed. They involved modulation of the amount of retinoids in the eye by targeting the cellular uptake of atROL by the RPE cells. The founders of this class of prospective drugs are inhibitors of serum retinol-binding protein (RBP4), namely, A1120, BPN14136, and their derivatives. They were examined in mouse models of Stargardt disease^46−48^ and two nonretinoid RBP4 antagonists advanced to clinical trials. Tinlarebant is being evaluated for the treatment of adolescent Stargardt disease in phase 3 (U.S. National Library of Medicine clinical trials database identifier—NCT05244304,). STG-001 was examined in a phase 2a clinical trial, showing a favorable safety profile with the exception of cases of delayed dark adaptation and night blindness in adult patients (NCT04489511).

An unconventional and innovative strategy, currently under commercial development and undergoing phase 2 clinical trials (NCT02402660), involves the inhibition of A2E biosynthesis through the administration of deuterated (C20-d3) retinoids.^49,50^ This approach capitalizes on the pronounced isotopic effect observed during the dimerization of retinal in the presence of a deuterated substrate. This phenomenon has yielded a deceleration in the rate of bis-retinoid accumulation, thereby leading to a subsequent attenuation in the progression of RPE atrophic changes in a mouse model of Stargardt disease.^51^

A more recent approach that alleviates potential side effects related to lowering RBP4 plasma levels is the inhibition of intracellular atROL transport. The first-in-class drug candidate that could be used for this purpose is abn-CBD, which acts as a competitive inhibitor of CRBP1.^34^ Consistent with the physiological role of CRBP1, the administration of this inhibitor affected the transport of atROL between the photoreceptor and RPE cells, which in turn resulted in delayed regeneration of the visual chromophore. The potential of targeting CRBP1 using this compound was further tested for light-induced retinal damage, protecting against this condition in albino mice.^34^ These preliminary data spearheaded our attempts to expand the search for alternative compounds that could potentially serve as high-affinity inhibitors of CRBP1.

The data presented in this study provide detailed insight into the binding capabilities of CRBP1 and the interaction profile of the identified competitive inhibitors. The newly identified chemical scaffolds for CRBP1 inhibitors serve as the primary platforms for further medicinal chemistry modifications. Due to their ability to displace endogenous atROL, their drug-like properties, and no history of medical use, the identified compounds are ideal for structure-based optimization. However, any rational attempts to improve the pharmacodynamic properties of CRBP1 inhibitors must consider the unique structural dynamics of the protein in its apo and holo form. Therefore, the geometric fit and specific interactions of potential inhibitors with the binding pocket are only prerequisites for a high-affinity interaction. Equally important is the ability of a ligand to stabilize the closed conformation of the portal region. It is tempting to speculate that the initial interaction of a CRBP1 ligand with the conformationally flexible portal region allows the ligand to slip into the binding cavity. Once inside, interactions of the bound compound with the residues of the portal region stabilize its conformation, effectively trapping the ligand inside the protein. In physiological conditions, these interactions appear to be critical for the discrimination between retinoids and other endogenous hydrophobic compounds and most likely contribute to the binding specificity of CRBP1. However, this mechanism can potentially be utilized to design potent inhibitors of CRBP1, as exemplified by abn-CBD. The only polar interactions between abn-CBD and CRBP1 occur within the portal region. Yet, they are sufficient to render an apparent binding affinity in the low nanomolar range.^34^ In contrast, despite the formation of networks of hydrogen bonding deeper in the binding site, inhibitors 1–6 revealed *K*_i_ values nearly three orders of magnitude higher (Table 1 and Figures 2 and 3). This decline in the binding affinities can be attributed to much weaker interactions with the entry portal region of CRBP1, which in turn results in an increased rate of ligand dissociation from the binding pocket. It is worth mentioning that the role of the portal region in ligand binding is not unique to CRBP1. Similar conformational changes upon atROL binding were observed for CRBP2 or its rat and zebrafish orthologs.^37,40,52,53^ Thus, the ligand locking mechanism that involves the portal region is preserved in the other members of the CRBP protein family.

Another contributing factor that may impact the efficacy of CRBP1/ligand integration is the overall hydrophobic nature of the interaction. The exact mechanism through which CRBPs acquire atROL within cells remains elusive. Nonetheless, it is highly plausible that lipophilic interactions with the hydrophobic region proximate to the portal site play a pivotal role in initiating protein–ligand interactions, subsequently followed by the establishment of specific hydrogen bonding deeper within the binding pocket. In this context, the compound abn-CBD, distinguished as the most lipophilic with a log *P* value of 6.5, could potentially encounter a lower energetic barrier while accessing the CRBP1 binding site compared to other significantly more polar inhibitors assessed in this study (Table 1).

The rational development or improvement of biologically active molecules relies predominantly on understanding their interactions with protein scaffolds. Most currently used methods for lead compound optimization are based on docking and scoring systems, where the binding mode of the ligand is predicted, followed by an estimation of the free energy of binding.^54^ More precise methodologies involve Monte Carlo simulations, which require significant computational power for extensive free energy sampling.^55^ However, most of these drug optimization approaches depend on the use of static protein–ligand complex structures acquired from the Protein Data Bank (PDB) or molecular docking, ignoring the potential flexibility and dynamics of the complexes.^56^ As a result, the prediction accuracy for high-affinity compounds and the overall success rate of structure-based computer-aided drug design and *in silico* screening remain relatively low. The function of macromolecular receptors is not only determined by their structures but also by their dynamics, as exemplified by our search for CRBP1 inhibitors. Therefore, disregarding the conformational flexibility of protein scaffolds within or outside a binding cavity can result in false or misleading drug optimization efforts. Importantly, this limitation of commonly used algorithms for docking or drug binding optimization is being addressed in the most recent computer-aided drug design methodologies. Novel approaches integrate information about the conformational dynamics of drug targets in the form of MD trajectories and combine them with machine learning algorithms to improve the performance of binding affinity predictions.^57−60^ Although the results of these attempts are still protein-dependent,^61−64^ the incorporation of structural dynamics information is a key factor in improving the accuracy of binding affinity prediction.

In summary, we have identified new chemical scaffolds for competitive inhibitors of CRBP1, expanding the structural diversity of first-in-class drug candidates targeting this intracellular atROL carrier. Importantly, the outcome of this study further signifies the functional importance of the portal region, the conformational changes associated with ligand binding, and their contribution to binding affinities. Finally, our data provide a strong argument for the necessity of incorporating MD components into modern algorithms for predicting interactions between small molecules and macromolecules.

## Materials and Methods

### Chemicals and Reagents

4-[(1*R*,6*R*)-3-Methyl-6-prop-1-en-2-ylcyclohex-2-en-1-yl]-5-pentylbenzene-1,3-diol (abnormal cannabidiol, abn-CBD) was obtained from Cayman Chemical. The remaining compounds tested in this study are listed in Table 1 and were obtained from MolPort or Hit2Lead.

### Expression and Purification of Recombinant Human CRBP1

The synthetic cDNA sequence of human CRBP1 (hCRBP1) (Gen ID: 5947) including six additional His-residues at the C-terminus was purchased from ATUM. The protein was expressed and purified according to the established methodology described by Silvaroli et al.^35^

### Obtaining CRBP1 in Complex with atROL

To prepare the holo form of human CRBP1, 3 mg mL^–1^ of purified apo CBRP1 in 10 mM Tris-HCl, pH 8.0, and 5% glycerol (v/v) was incubated on ice for 15 min with ∼4 molar excess of atROL (Toronto Research Chemicals) in ethanol. The solution was then diluted 10x with 10 mM Tris-HCl, pH 8.0, and centrifuged (36,000*g*, 20 min, 4 °C). The supernatant was loaded onto a 5 mL HiTrap Q HP column (Cytiva) and eluted in a linear gradient of 1 M NaCl in 10 mM Tris-HCl, pH 8.0. The efficiency of loading of CRBP1 with atROL was assessed by recording the UV/vis spectra. The characteristic absorbance spectra of the CRBP1/atROL complex revealed maxima at 282 nm, representing the protein scaffold and triple maxima at 332, 348, and 365 nm corresponding to atROL with a *A*_350_/*A*_280_ ratio of ∼1.6, as reported previously.^35,65^

### Determination of the Inhibition Constant Using Fluorescence atROL-Replacement Assays

The apparent inhibition constant (*K*_i_) of putative CBRP1 inhibitors was assessed by recording changes in the fluorescence spectra of atROL-CRBP1 in the presence of tested compounds. All measurements were conducted in 67 mM phosphate-buffered saline buffer, pH 7.4, containing 5% glycerol (v/v) using a LS55 spectrofluorometer (PerkinElmer Life Sciences) at RT. Holo CRBP1 excited at 285 nm emits fluorescence with maxima at 350 and 480 nm due to fluorescence resonance energy transfer (FRET) between the tryptophan residues of CRBP1 and the retinoid moiety. The replacement of atROL by an alternative compound diminishes FRET, which results in the increase of fluorescence intensity at 350 nm (corresponding to the protein scaffold) and a concomitant decrease at 480 nm (fluorescence of the retinoid moiety). For the titration, the compounds under investigation, dissolved in acetonitrile, were added at final concentrations ranging between 0 and 10 μM in the cuvette. The corresponding changes in protein fluorescence were used to plot titration curves and calculate the *K*_i_ values using SigmaPlot 11 software (Systat Software).

### High-Throughput Screening (HTS) for Non-retinoid Ligands of CRBP1

The fluorescence binding assay described above was, in principle, applied for the HTS in a 384-well plate format. Each well contained 50 μL of 20 mM Tris/HCl, pH 7.4, and 1 μM holo CRBP1. A small-molecule library with drug-like characteristics (ChemBridge NT1299 library) was delivered in dimethyl sulfoxide (DMSO) to each well to a final concentration of 5 μM. Well-plates were incubated at RT for 10 min and analyzed in a SynergyNeo2 microplate reader (Agilent) by exciting samples at 285 nm and recording fluorescence intensities at 350 nm (CRBP1) and 480 nm (atROL). abn-CBD, a previously identified ligand of CRBP1,^34^ was used as a positive control for the HTS. Wells that contained holo CRBP1 and DMSO served as a negative control. HTS hits were characterized by changes in the fluorescence emission at 350 and 480 nm and were compared to those observed for abn-CBD. To analyze the outcome of the HTS, the following equation was applied for each of the measured compounds
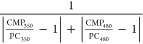
where CMP_350_—fluorescence signal at 350 nm for an examined HTS compound; CMP_480_—fluorescence signal at 480 nm for an examined HTS compound; PC_350_—fluorescence signal at 350 nm for the positive control (abn-CBD) ; and PC_480_—fluorescence signal at 480 nm for the positive control.

### Crystallization of CRBP1 in Complex with its Nonretinoid Ligands

CRBP1 in complex with potential inhibitors was crystallized by incubating apo protein at a concentration of 3 mg mL^–1^ with 300 μM of the tested compound in 10 mM Tris-HCl buffer, pH 8.0, 150 mM NaCl on ice for 15 min. Sitting-drop crystallization plates were set up by mixing 1 μL of the protein sample with 1 μL of the crystallization buffer composed of 0.1 M Bis–Tris, pH 5.5, and 25% poly(ethylene glycol) 3350 (w/v). The crystals were grown at RT for 5–7 days before being harvested and flash-frozen in liquid nitrogen.

### X-ray Data Collection, Processing, and Model Building

For the collection of X-ray diffraction patterns, the Advanced Photon Source beamlines NE-CAT 24-ID-C and 24-ID-E were used. Integrating and scaling the data was conducted with the XDS^66,67^ and CCP4 platforms.^68^ The protein structures were solved using molecular replacement with PHASER_MR^69^ and a high-resolution template of human CRBP1 (PDB 5HBS).^35^ Further manual correction of the initial model was conducted using WinCoot^70^ and the refinement was acquired with PHENIX.^71^ The geometry of the refined model was verified using the MolProbity server.^72^ The final atomic coordinates and structure factors for all structures described in this report were deposited in the RCSB Protein Data Bank. The accession codes, as well as the data collection and refinement statistics, are summarized in Table S1. For the crystallographic ensemble refinement,^41^ the following settings were used: fraction of atoms for TLS fitting—0.8; temperature-controlled X-ray weight—5 K; simulation temperature—300 K; relaxation time—value determined based on the data set resolution. The number of protein models in the ensembles was not arbitrarily limited. Protein models represented in the figures were prepared with the CHIMERA software package version 1.16.^73^

### Calculations of Average Crystallographic B-Factors and Distribution of Anisotropy within Refined Models

The averages of the equivalent isotropic crystallographic *B*-factors (*B*_eq_) were normalized with “*z*-score normalization” methodology where *B*_eqX-*z*-score(i)_ is the normalized *z*-score for residue X in structure i, *B*_eqX(i)_ is the equivalent isotropic *B*-factor for residue X, ⟨*B*_eq(i)_⟩ is the average residue equivalent isotropic *B*-factor for structure i, and *S*_(i)_ is the corresponding standard deviation among atoms in the structure^42^
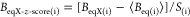


### Amide Hydrogen/Deuterium (H/D) Exchange Mass Spectrometry

The uptake of deuterium for apo and ligand-bound CRBP1 was determined to assess the effect of the ligand binding on the structural flexibility of the protein scaffold. All sample preparations were conducted on ice with minimal exposure to air. The test compound was added in 5-fold molar excess to apo CRBP1 and incubated on ice for 10 min. The H/D exchange buffer was composed of 10 mM Tris/HCl, pH 7.4, 50 mM NaCl in 99.9% D_2_O. Twenty μg of holo CRBP1 (2 μL of protein solution) was added to 78 μL of the exchange buffer and incubated for 1, 2, 5, and 10 min. The H/D exchange reaction was quenched by lowering the pH to 2.0 with 10 μL of 1% formic acid. The protein was digested with 10 μL of 8 mg mL^–1^ freshly prepared pepsin (Worthington) for 2 min on ice. The resulting peptides were loaded onto a C4 (2.1 × 50 mm^2^, Thermo Scientific) column using a temperature-controlled Agilent 1100 autosampler and binary pump (Thermo Scientific). Peptides were eluted in a gradient of acetonitrile in water (2–100%) over 20 min at a flow rate of 0.2 mL min^–1^. The eluent was directed into an LTQ Velos linear trap mass spectrometer (Thermo Scientific) via an electrospray ionization source operated in positive ion mode. Peptides resulting from pepsin digestion were identified by tandem mass spectrometry (MS^2^). Raw data in the form of the relative signal intensity as a function of *m*/*z* were extracted with Xcalibur version 2.1.0 (Thermo Scientific) and a deconvolution procedure was performed with H/X-Express 2.^74^ The average deuterium content of each fragment ion was calculated by using the centroid of its isotopic cluster. The extent of the H/D exchange was color-coded based on the maximal observed deuterium uptake and represented as a percentage of total theoretic uptake or change in the percentage of deuterium uptake between apo and holo forms of CRBP1.

### Molecular Dynamics (MD) Simulation

MD simulations for all nine systems (atROL, abn-CBD, inhibitors 1–6, and apo CRBP1) were parametrized and prepared using CHARMM-GUI.^75^ Each system was explicitly solvated with a 10 Å buffer between the protein and the edge of the TIP3P water box and neutralized via the addition of KCl at a concentration of 0.15 M, resulting in simulation systems containing ∼21,000 atoms each. All nine CRBP1 structures were simulated in NAMD 2.13 using the CHARMM36 M force field.^76,77^ Systems were first minimized over 10,000 steps of conjugate gradient and equilibrated over 1 ns of NVT simulation. During equilibration, the system was heated to 303.15 K and heavy-atom positional restraints were progressively released over the course of the simulation. Langevin Dynamics was used to control the temperature, a 2 fs time step was used, and a 12 Å cutoff was used for nonbonded interactions. Classical MD production runs were performed in an NPT ensemble at 303.15 K and 1 atm controlled using Langevin dynamics and Nose–Hoover Langevin piston pressure control, respectively. Hydrogen mass repartitioning was used to reweight hydrogen atoms allowing for a 4 fs time step,^78^ and a 12 Å cutoff was used for nonbonded interactions. Each system was simulated for 500 ns in duplicate, resulting in a total simulation time of 9 μs. MD trajectories were analyzed using CPPTRAJ form AmberTools23.^79,80^ The trajectories were first autoimaged and aligned via the β strands of CRBP1 (residues 6–14, 39–45, 48–54, 60–65, 70–73, 81–89, 92–98, 105–111, 114–121, 124–133). Once aligned, heavy-atom root-mean-square deviation (RMSD) values (compared to the first frame) without fitting were calculated for the ligand, protein, and portal loops, and RMSF values were calculated for per-residue fluctuations and calculation of *B*-factors for visualization. Trajectories were visualized using both Chimera and Visual Molecular Dynamics.^73,81^

## Supporting Information Available

Additional experimental data organized in tables and figures: crystallographic tables featuring information about X-ray diffraction data collection and structure refinements; outcomes of structure–function relationship tests conducted on CRBP1 inhibitors, electron density maps, and comprehensive insights into the interactions within the binding pocket for CRBP1 inhibitors; and analysis of binding selectivity, along with the findings from molecular dynamics (MD) simulations (DOCX)

## Accession Codes

The structure factor files and the corresponding models of CRBP1 complexes with nonretinoid inhibitors described in this article have been deposited in PDB under the following accession numbers: 8GD2, 8GDM, 8GEM, 8GEU, 8GEV, and 8GEY.

## Abbreviations

11cRAL: 11-*cis*-retinal
A2E: pyridinium-ethanolamine
atRAL: all-*trans*-retinal
abn-CBD: abnormal cannabidiol
AMD: age-related macular degeneration
atROL: all-*trans*-retinol
*B*_*eq*_: equivalent isotropic crystallographic *B*-factors
CRBP1: cellular retinol-binding protein 1
CRBP2: cellular retinol-binding protein 2
CRBPs: cellular retinol-binding proteins
FRET: fluorescence resonance energy transfer
H/D: hydrogen/deuterium
HTS: high-throughput screen
*K*_i_: inhibition constant
*k*_off_: dissociation rate constant
LC/MS: liquid chromatography/mass spectrometry
MD: molecular dynamics
MS: mass spectrometry
PDB: Protein Data Bank
RALdi: retinaldehyde dimer
RBP4: serum retinol-binding protein
RMSD: root-mean-square deviation
RMSF: per-residue root-mean-square fluctuation
RPE: retinal pigment epithelium
RPE65: retinoid isomerase or retinal pigment epithelium-specific protein 65 kDa
WT: wild-type
