# Supplementary material for: Discovery of Nonretinoid Inhibitors of CRBP1: Structural and Dynamic Insights for Ligand-Binding Mechanisms
Source: ACS Chem Biol. 2023 Sep 15;18(10):2309–23. doi: 10.1021/acschembio.3c00402 (PMC10591915; doi:10.1021/acschembio.3c00402)
Supplement: Supplementary file 1 — cb3c00402_si_001.docx [file cb3c00402_si_001.docx]

**SUPPORTING INFORMATION**

**Discovery of Non-Retinoid Inhibitors of CRBP1: Structural and Dynamic Insights for Ligand Binding Mechanisms**

Jacqueline Plau^1^, Christopher E. Morgan^1,2^, Yuriy Fedorov^3^, Surajit Banerjee^4,5^, Drew J. ^Adams3,6^, William S. Blaner^7^, Edward W. Yu^1,8^, and Marcin Golczak^1,8*^

^1^Department of Pharmacology, ^3^Small Molecule Drug Development Core Facility, ^6^Department of Genetics, ^8^Cleveland Center for Membrane and Structural Biology, School of Medicine, Case Western Reserve University, Cleveland, Ohio, 44124, United States;

^2^Department of Chemistry, Thiel Collage, Greenville, Pennsylvania, 16125, United States;

^4^Department of Chemistry and Chemical Biology, Cornell University, Ithaca, New York, 14850, United States;

^5^Northeastern Collaborative Access Team, Argonne National Laboratory, Argonne, Illinoi, 60439, United States;

^7^Department of Medicine, College of Physicians and Surgeons, Columbia University, New York, 10032, New York, United States.

**Keywords**: cellular retinol-binding protein, *Rbp1*, CRBP1, vitamin A, retinol, retinoid metabolism

**Running title**: Inhibitors of CRBP1

*To whom the correspondence should be addressed: Marcin Golczak, Ph.D., Department of Pharmacology, School of Medicine, Case Western Reserve University, 10900 Euclid Ave, Cleveland, Ohio 44106, USA; Phone: 216–368–0302; E–mail: [mxg149@case.edu](mailto:mxg149@case.edu).

**Table S1** – X-ray data collection and refinement statistics.

| **Protein**  **Ligand** | **CRBP1**  **1** | **CRBP1**  **2** | **CRBP1**  **3** | **CRBP1**  **4** | **CRBP1**  **5** | **CRBP1**  **6** |
| --- | --- | --- | --- | --- | --- | --- |
| **PDB code**  **Beam line**  Wavelength (Å)  **Data collection** | **8GD2**  24-ID-C  0.9791 | **8GEM**  24-ID-C  0.9791 | **8GEV**  24-ID-C  0.9791 | **8GEY**  24-ID-C  0.9791 | **8GDM**  24-ID_E  0.9792 | **8GEU**  24-ID_E  0.9795 |
| Space group | *P*2_1_2_1_2_1_ | *P*2_1_2_1_2_1_ | *P*2_1_2_1_2_1_ | *P*2_1_2_1_2_1_ | *P*2_1_2_1_2_1_ | *P*2_1_2_1_2_1_ |
| Cell dimensions |  |  |  |  |  |  |
| *a*, *b*, *c* (Å)  α, β, γ (°) | 37.33, 37.69, 101.54  90.00, 90.00, 90.00 | 37.74, 40.06, 93.67  90.00, 90.00, 90.00 | 37.30, 38.67, 93.70  90.00, 90.00, 90.00 | 37.48, 43.02, 89.22  90.00, 90.00, 90.00 | 37.35, 39.01, 94.03  90.00, 90.0, 90.00 | 37.15, 37.88,  101.97  90.00, 90.0, 90.00 |
| Resolution (Å) | 101.54-1.13^1^  (1.15-1.13)^2^ | 46.83-1.55^1^  (1.58-1.55)^2^ | 93.70-1.85^1^  (1.89-1.85)^2^ | 43.02-1.30^1^  (1.32-1.30)^2^ | 94.03-1.80^4^  (1.84-1.80)^2^ | 37.88-1.47^1^  (1.49-1.47)^2^ |
| *R*_merge_ (%)  *R_pim_* (%) | 4.3 (30.7)^2^  2.3 (20.2)^2^ | 4.7 (9.67)^2^  1.8 (41.3)^2^ | 14.5 (95.8)^2^  10.5 (71.1)^2^ | 11.0 (97.9)^2^  4.5 (51.2)^2^ | 8.8 (85.4)^2^  4.4 (4.1)^2^ | 10.7 (70.0)^2^  4.6 (30.5)^2^ |
| *I*/σ*I*  *CC(1/2)* | 20.6 (5.0)^2^  0.99 (0.96)^2^ | 17.3 (1.9)^2^  1.00 (0.66)^2^ | 7.7 (2.5)^2^  0.99 (0.55)^2^ | 11.4 (2.0)^2^  0.99 (0.67)^2^ | 9.1 (1.8)^2^  0.99 (0.76)^2^ | 20.6 (4.2)^2^  0.99 (0.76)^2^ |
| Completeness (%) | 94.2 (67.7)^2^ | 99.9 (83.6)^2^ | 99.7 (99.8)^2^ | 99.4 (96.9)^2^ | 97.8 (99.2)^2^ | 98.8 (94.5)^2^ |
| Redundancy | 5.8 (3.4)^2^ | 6.4 (6.4)^2^ | 4.9 (5.2)^2^ | 5.7 (3.5)^2^ | 3.8 (4.0)^2^ | 5.8 (5.1)^2^ |
|  |  |  |  |  |  |  |
| **Refinement** |  |  |  |  |  |  |
| Resolution (Å) | 35.33-1.13 | 46.83 -1.55 | 46.85-1.85 | 38.75-1.30 | 36.04-1.80 | 34.91-1.47 |
| No. of reflections | 51,290 | 21,274 | 12,038 | 35,783 | 12,960 | 24,780 |
| *R*_work_/*R*_free_ (%)  No. of atoms  Protein  Ligand  Water  Mean *B*-factor (Å^2^)  Protein  Ligand  Water  R.m.s. deviations  Bond lengths (Å)  Bond angles (°)  **Validation**  Ramachandran  Favored/outliers (%)  Rotamer outliers (%)  Clash score | 16.2/18.4  1,577  1,224  26 (Z5H)^3^  14 (BTB)^3^  313  13.7  18.4 (Z5H)^3^  16.6 (BTB)^3^  29.4  0.015  1.654  97.8/0  0.75  2.8 | 21.4/25.6  1,310  1,124  28 (ZDF)^3^  158  35.4  40.3 (ZDF)^3^  43.0  0.018  1.505  97.7/0  0.81  4.9 | 18.6/23.3  1,299  1,139  28 (ZDK)^3^  132  24.1  34.2 (ZDK)^3^  32.4  0.018  1.522  97.8/0  0  5.2 | 16.3/18.2  1,522  1,171  24 (ZE2)^3^  14 (BTB)^3^  313  13.0  16.3 (ZE2)^3^  26.0 (BTB)  26.0  0.017  1.612  98.5/0  0  2.1 | 18.3/21.3  1,324  1,144  28 (ZA6)^3^  145  35.9  44.9 (ZA6)^3^  44.9  0.005  0.944  98.0/0  0  4.4 | 18.2/20.3  1,452  1,155  27 (ZCF)^3^  14 (BTB)  253  13.5  19.6 (ZCF)^3^  19.8 (BTB)  24.2  0.011  1.216  79.6/0  0  6.4 |
|  |  |  |  |  |  |  |

^1^Data set was collected on a single crystal

^2^Highest-resolution shell is shown in parentheses

^3^Ligand’s accession code





**Figure S1** – *List of initial HTS hits that did not yield macromolecule crystals in complex with CRBP1.* Protein crystallization served as a stringent secondary screening method to validate the authentic interaction of potential ligands with the protein.





**Figure S2** – *Outcome of a partial exploration into the structure-function relationship of CRBP1 inhibitors.* Notably, chemical alterations implemented in compounds denoted by an asterisk resulted in the suppression of binding to CRBP1.

**
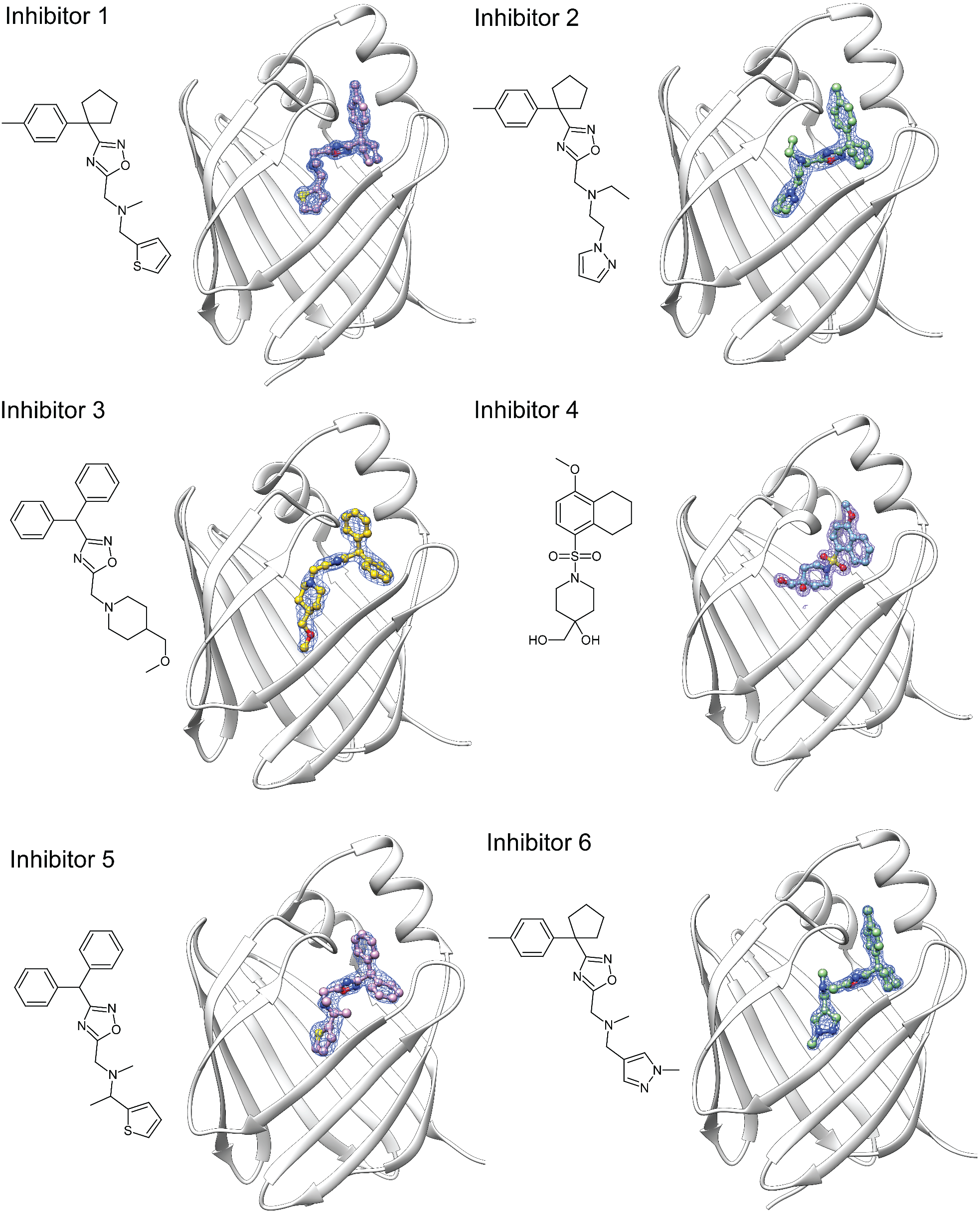
**

**Figure S3** – *Crystal structures of CRBP1 in complex with its non-retinoid inhibitors*. Individual compounds in the binding pocket of the protein are indicated with ball-and-stick models. The blue mesh corresponds to the 2F_o_ – F_c_ electron density map contoured at 1.2 σ.


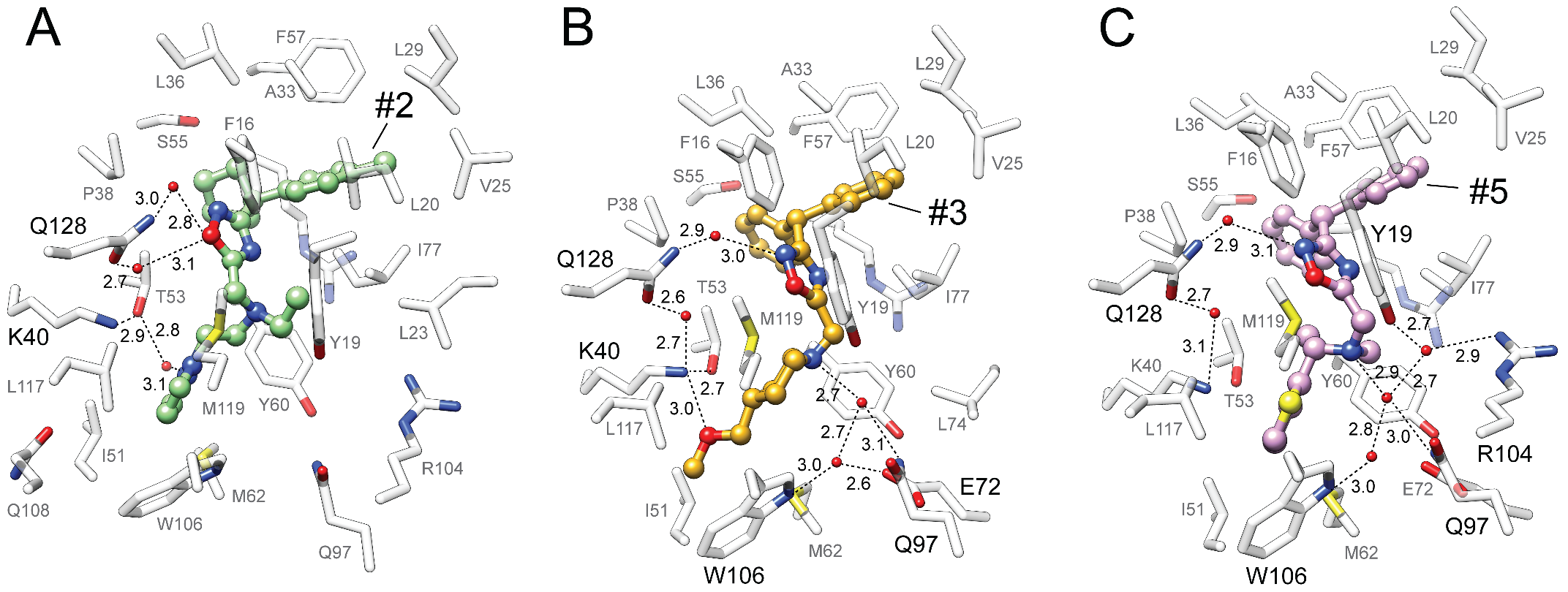


**Figure S4** – *Details of the interaction of selected CRBP1 inhibitors in the binding pocket.* Panels A, B, and C correspond to inhibitors 2, 3, and 5, respectively (PDB #8GEM, #8GEV, and #8GDM). Ordered water molecules are shown as red spheres; dashed lines indicate hydrogen bonds. Distances are shown in angstroms.


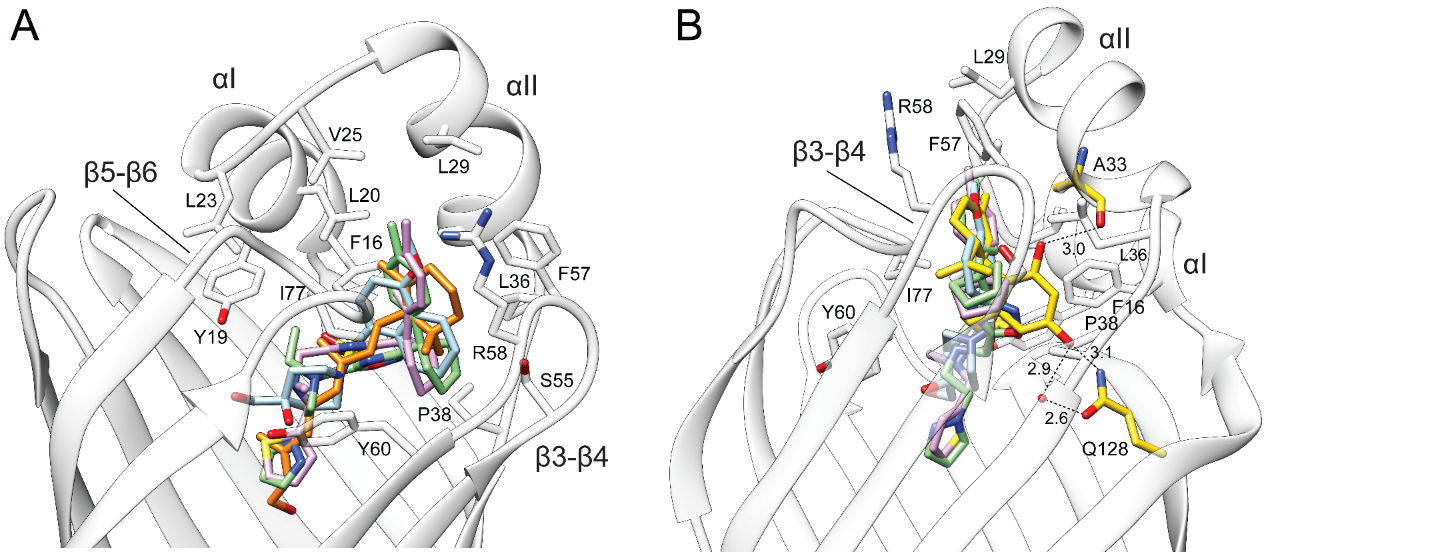


**Figure S5** – *Interaction of the CRBP1 inhibitors with the portal region of the protein.* (A) Overlap of atROL (orange) and inhibitors 1 (purple), 2 (green), and 4 (blue). Side chains of residues within 4 Å distance from the ligands are marked. (B) Details of the interaction of abn-CBD (yellow) with the portal region of CRBP1. Unlike other inhibitors, abn-CBD forms a hydrogen bond with the main chain of A33 stabilizing the conformation of α-helix II. An additional interaction occurs between the inhibitor and the side chain of Q128. Dashed lines indicate hydrogen bonds. Distances are shown in angstroms.

**
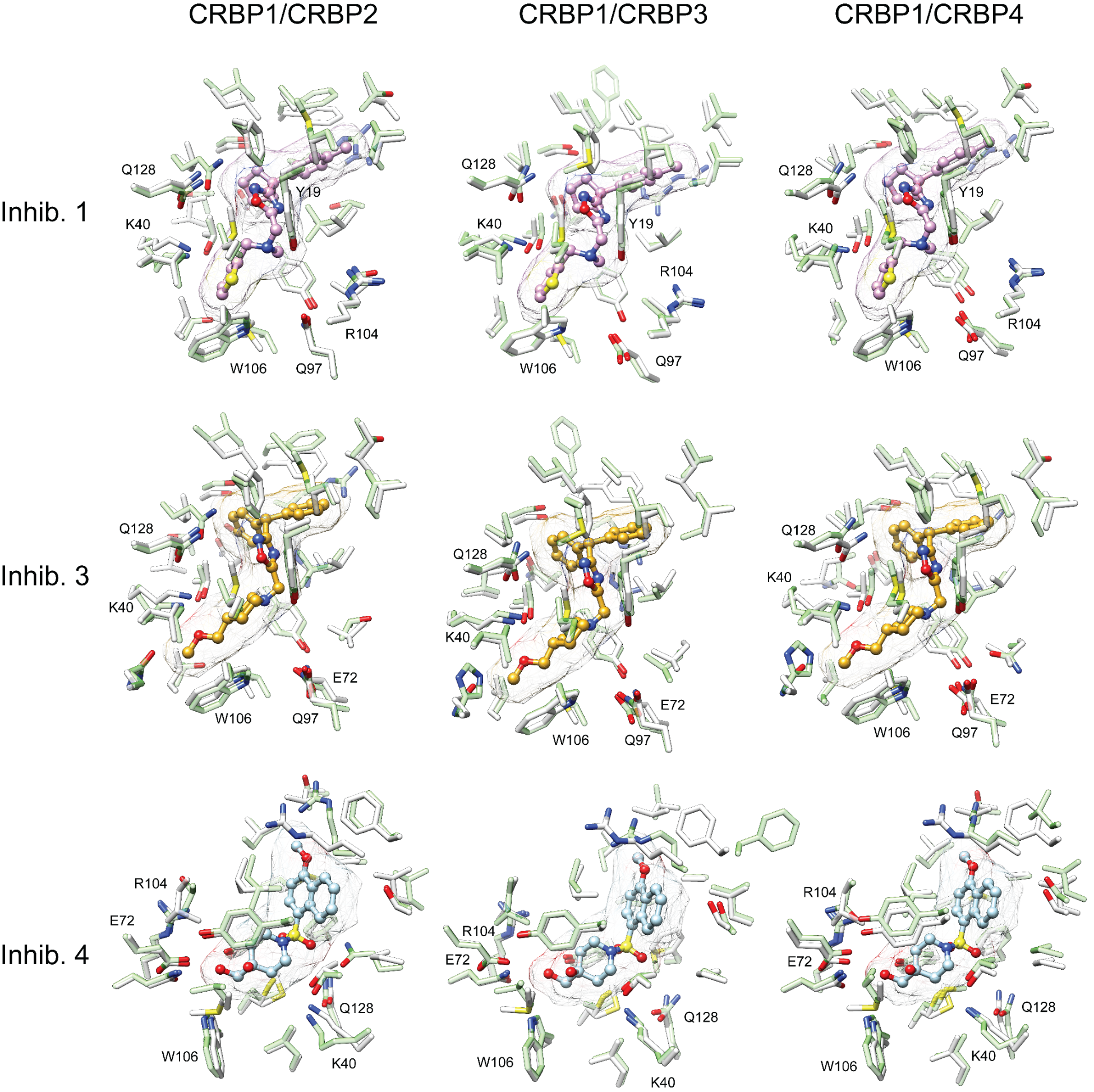
**

**Figure S6** – *Depiction of structural foundations for the interactions between the newly identified inhibitors of CRBP1 and other members of the CRBP protein family.* Close-up view of the ligand binding site within CRBP1 (depicted in white) complexed with inhibitors 1 (in pink), 3 (in yellow), or 4 (in blue). These structures are superimposed onto the corresponding structures of CRBP2 (PDB #6BTH), CRBP3 (PDB #6E5W), and CRBP4 (PDB #6E6K), which are represented in green. A detailed analysis of the binding pockets within CRBP1 and its closely related proteins did not reveal any alterations in architecture that could obstruct the binding of the examined inhibitors. Moreover, the essential amino acids engaged in polar interactions with these ligands (indicated by their type and position within the protein sequence) are consistently conserved across all human CRBPs.


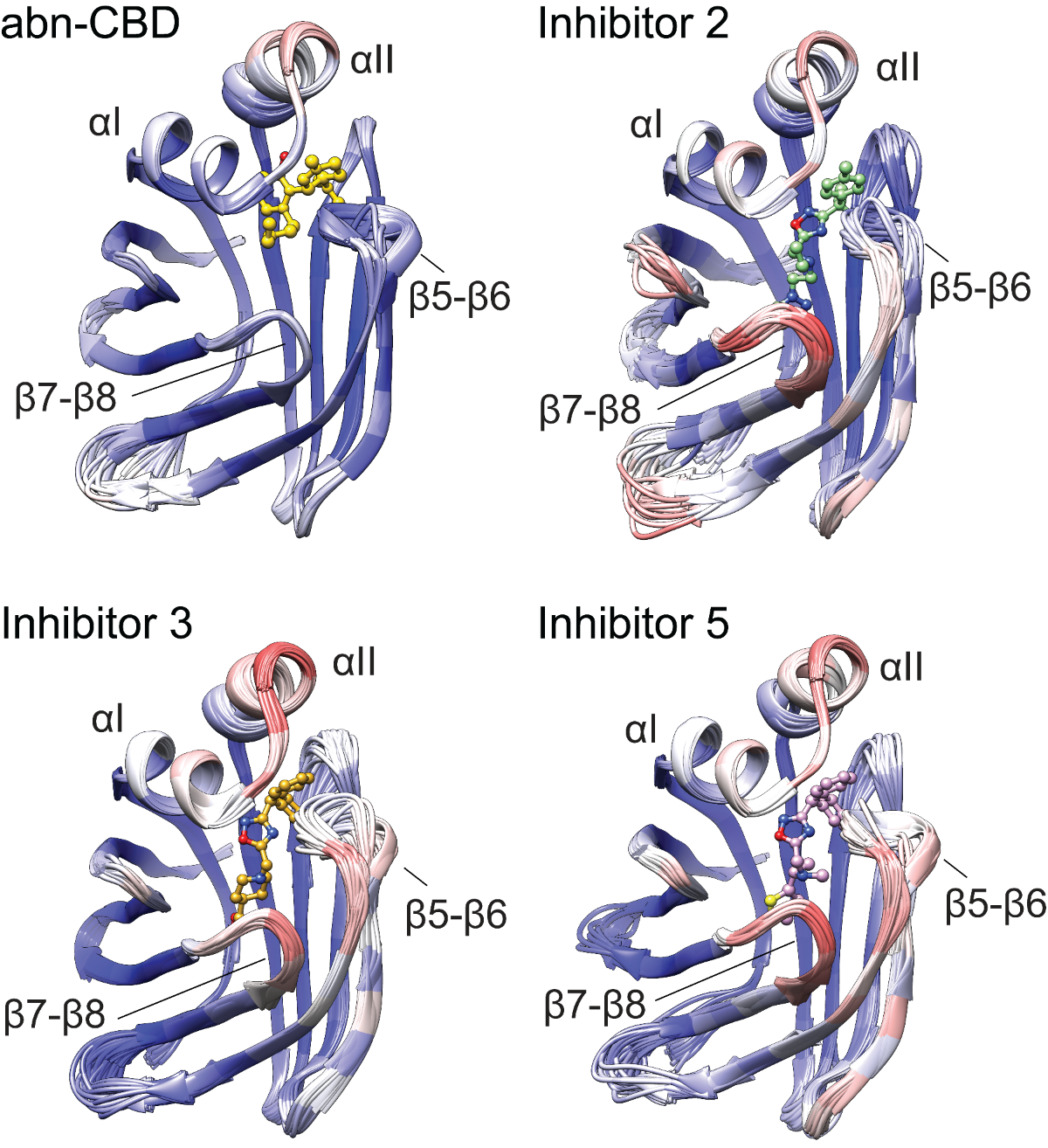


**Figure S7** – *Comparison of structural dynamics of CRBP1 upon binding of its non-retinoid inhibitors.* The structures represent the ensemble refinement of the crystallographic structures of CRBP1 in the apo and inhibitor-bound states. The color scheme represents average *B* -factors per residue in which the highest values are marked red and the lowest in blue. Superimposition of individual structures of the assemblies revealed increased structural flexibility of the portal region in CRBP1 when bound to inhibitors 2, 3, and 5 as compared to abn-CBD.


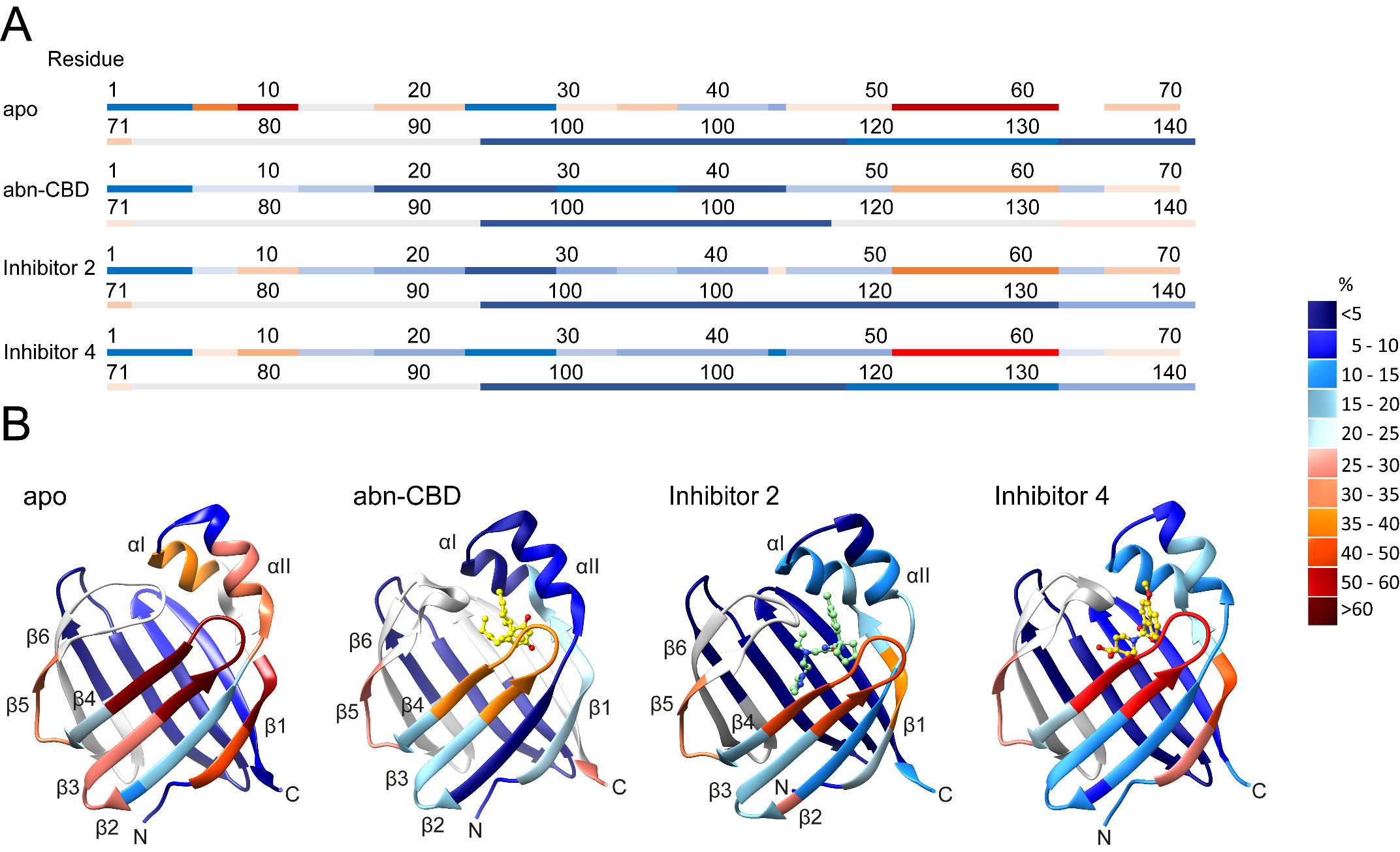


**Figure S8** – *Hydrogen/deuterium (H/D) exchange mass spectrometry analysis of the CRBP1 structure in apo and inhibitor-bound states.* The color scheme indicates the percentage of maximum uptake as an indicator of structure stability. (A) Color coded sequences of human CRBP1 indicate the relative deuterium uptake for individual regions of the protein. (B) Visualization of H/D exchange through the protein structure. Apo CRBP1 revealed the highest structural flexibility as indicated by the efficiency of deuterium uptake. Binding of the high-affinity inhibitors such as abn-CBD dramatically lowers deuterium uptake, particularly for α-helix I/II and β-hairpin 2-3. Importantly, the magnitude of H/D exchange depends on the type of ligand and correlates with its overall affinity.


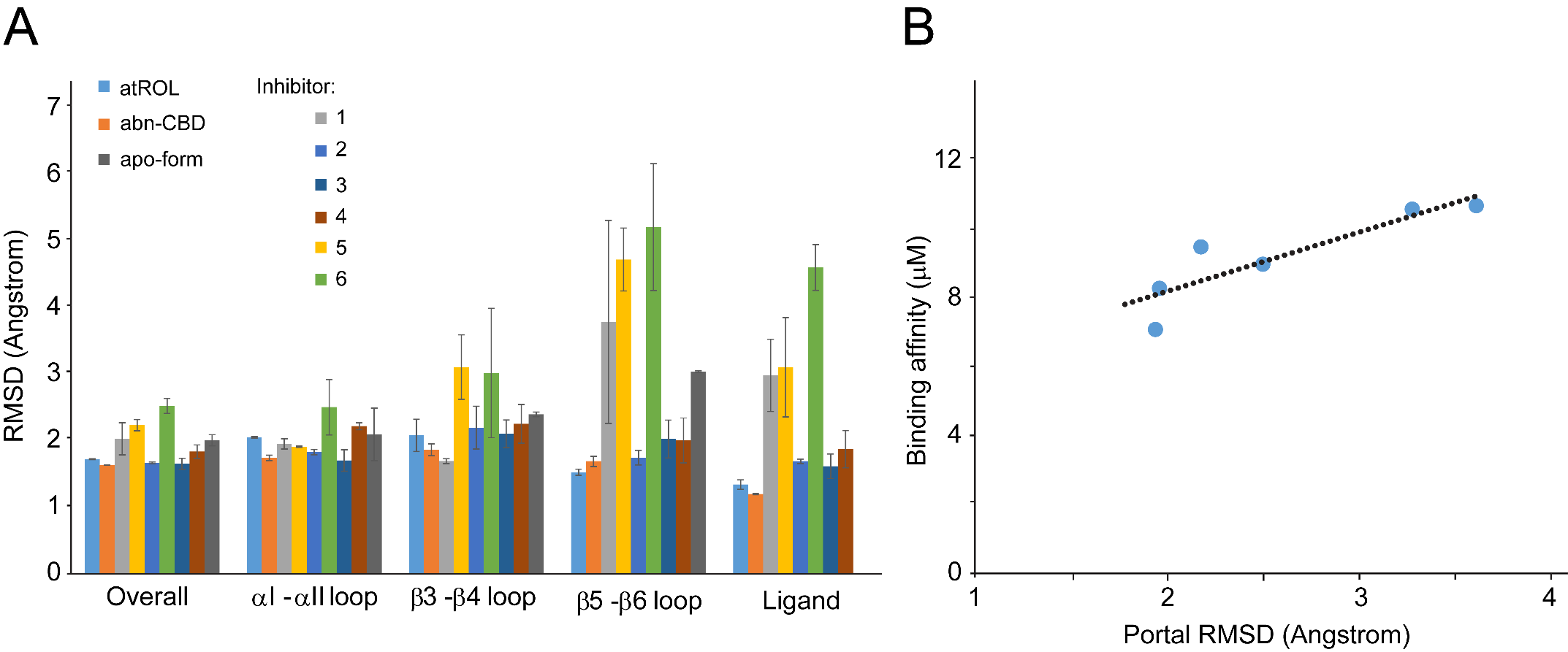


**Figure S9** *– RMSD analysis of MD trajectories for the portal region of CRBP1.* (A) Bar plot shows RMSD values for the overall CRBP1 protein (residues 6-133), αI-αII (residues 18 to 36), the β3-β4 loop (residues 53 to 60), the β5-β6 loop (residues 73 to 81) and the inhibitors of interest for all nine simulation applied in this study. (B) Correlation of the average RMSD value of the portal region (αI-αII, and loops β3-β4 and β5-β6) with measured binding affinity for the six tested inhibitors. Data is correlated with an R^2^ value of 0.78.

**Table S2** – *Average RMSD values from MD simulations*.*

|  | Overall | αI-αII loop | β3-β4 loop | β5-β6 loop | Ligand |
| --- | --- | --- | --- | --- | --- |
| atROL | 1.72 ± 0.01 | 2.05 ± 0.01 | 2.08 ± 0.24 | 1.53 ± 0.05 | 1.34 ± 0.07 |
| abn-CBD | 1.64 ± 0.01 | 1.75 ± 0.04 | 1.87 ± 0.09 | 1.69 ± 0.08 | 1.20 ± 0.01 |
| inhibitor 1 | 2.03 ± 0.24 | 1.95 ± 0.08 | 1.69 ± 0.04 | 3.79 ± 1.52 | 2.99 ± 0.54 |
| inhibitor 2 | 1.67 ± 0.01 | 1.83 ± 0.04 | 2.20 ± 0.32 | 1.74 ± 0.11 | 1.69 ± 0.03 |
| inhibitor 3 | 1.66 ± 0.08 | 1.71 ± 0.16 | 2.11 ± 0.21 | 2.03 ± 0.29 | 1.62 ± 0.19 |
| inhibitor 4 | 1.84 ± 0.10 | 2.22 ± 0.05 | 2.26 ± 0.29 | 2.01 ± 0.34 | 1.88 ± 0.28 |
| inhibitor 5 | 2.24 ± 0.08 | 1.91 ± 0.01 | 3.11 ± 0.49 | 4.73 ± 0.47 | 3.11 ± 0.75 |
| inhibitor 6 | 2.53 ± 0.11 | 2.51 ± 0.42 | 3.02 ± 0.97 | 5.22 ± 0.95 | 4.61 ± 0.34 |
| apo protein | 2.01 ± 0.08 | 2.10 ± 0.40 | 2.40 ± 0.03 | 3.04 ± 0.01 | N/A |

*RMSD values were measured after alignment of β-sheets in CRBP1 (residues 6-14, 39-45, 48-54, 60-65, 70-73, 81-89, 92-98, 105-111, 114-121, 124-133). Measured values for the overall protein (residues 6-133), the αI-αII loop (residues 24 to 36), the β3-β4 loop (residues 53 to 60), the β5-β6 loop (residues 73 to 81) and the ligand of interest for all nine simulation systems studied are provided. Average RMSD ± SEM (standard error of the mean) are provided in angstroms.
